# Machine learning identifies MiRNA biomarkers and immune mechanisms in active tuberculosis

**DOI:** 10.1038/s41598-025-20112-8

**Published:** 2025-10-16

**Authors:** Zihan Cai, Chunxiao Huang, Yuyang Zhou, Lixian Wu, Shoupeng Ding

**Affiliations:** 1 Department of Medical Laboratory, Siyang Hospital, Siyang, 223700 China; 2 Oncology and Laboratory Immunology Research Innovation Center, Siyang Hospital, Siyang, 223700 China; 3 Department of Laboratory Medicine, Gutian County Hospital, Gutian, 352200 China; 4 Department of Microbiology and Immunology, School of Basic Medical Sciences, Dali University, Dali, 671000 China

**Keywords:** Tuberculosis, MicroRNA, Biomarker, Machine learning, Functional enrichment analysis, Computational biology and bioinformatics, Immunology, Microbiology, Biomarkers

## Abstract

Tuberculosis (TB), caused by *Mycobacterium tuberculosis* (Mtb), remains a major global public health threat. The rising prevalence of HIV/TB co-infection and multidrug-resistant tuberculosis (MDR-TB) has further intensified this challenge. This study aims to explore the role of microRNAs (miRNAs) in the immune response to Mtb infection and to identify potential miRNA biomarkers for active TB diagnosis using machine learning techniques. miRNA expression profiles were retrieved from the Gene Expression Omnibus (GEO) database (accession number: GSE70425). Differential expression analysis between active TB and latent TB infection (LTBI) patients was conducted using the “limma” package, with a screening threshold of |logFC| > 0.25 and *p*-value < 0.05. Key differentially expressed miRNAs (DE-miRNAs) were further refined using machine learning algorithms, including least absolute shrinkage and selection operator (LASSO) regression, support vector machine-recursive feature elimination (SVM-RFE), and Boruta. TargetScan was employed to predict miRNA target genes, and a regulatory network was visualized using Cytoscape. Subsequently, Gene Ontology (GO) and Kyoto Encyclopedia of Genes and Genomes (KEGG) pathway enrichment analyses were conducted to elucidate the functional roles of target mRNAs. Nine machine learning models were developed based on the selected miRNAs, and their predictive performance was assessed using metrics including AUROC, accuracy, sensitivity, specificity, positive predictive value (PPV), and negative predictive value (NPV). In vitro experiments were performed using THP-1 macrophages to establish an Mtb infection model. Cells were transfected with miR-3607-3p mimics and negative controls, followed by flow cytometry for apoptosis detection, Western blot for protein expression analysis, and Quantitative Real-Time PCR (qRT-PCR) for validation of apoptosis-related gene expression. A total of 72 differentially expressed miRNAs were identified. Key miRNAs, including hsa-miR-3607-3p, hsa-miR-148b, and hsa-miR-519e, were identified through multiple machine learning methods, with hsa-miR-3607-3p emerging as the primary candidate miRNA. The nine machine learning models exhibited robust predictive performance in both the training and test sets. PPI network and functional enrichment analyses indicated that the target genes of hsa-miR-3607-3p are primarily associated with cell growth and apoptosis-related pathways. In vitro experiments further suggested that hsa-miR-3607-3p may modulate the apoptotic response of THP-1 cells to Mtb infection via a caspase-dependent mechanism. miR-3607-3p was upregulated in active TB patients and may modulate the apoptosis of THP-1 cells during Mtb infection via a caspase-dependent pathway. The mechanism of this miRNA offers preliminary insights into the immune regulation of tuberculosis. miR-3607-3p may serve as a potential biomarker for the early diagnosis and intervention of active TB; however, its clinical applicability necessitates further validation through larger sample sizes and multicenter studies.

**Supplementary Information:**

The online version contains supplementary material available at 10.1038/s41598-025-20112-8.

## Introduction

Tuberculosis (TB), caused by infection with *Mycobacterium tuberculosis* (Mtb), remains one of the most significant infectious diseases threatening global public health. As reported in the 2024 Global Tuberculosis Report by the World Health Organization, 1.25 million individuals worldwide died from TB in 2023, making it the second leading cause of death after COVID-19^1,2^. The rising incidence of HIV/TB co-infections, coupled with the growing burden of multidrug-resistant TB (MDR-TB), has intensified the challenge of controlling TB, particularly in low-resource settings^3,4^.

Mtb is an intracellular pathogen that facilitates its long-term survival by modulating the host immune system. The host immune system plays a pivotal role in clearing *Mtb* infection, with macrophages functioning as the primary host cells responsible for pathogen elimination via mechanisms such as apoptosis, autophagy, phagolysosome maturation, and anti-inflammatory responses^5–9^. However, Mtb can evade immune surveillance and persist within host cells, resulting in latent TB infection (LTBI), which may progress to active TB (ATB) in approximately 10% of individuals. Although antibiotics remain the cornerstone of TB treatment, the emergence of drug-resistant strains and the complexity of treatment present significant challenges to disease control^10–12^.

In recent years, studies have shown that *Mycobacterium tuberculosis* (Mtb) not only disrupts host protein networks but also modulates immune responses by regulating microRNAs (miRNAs)^13,14^. miRNAs are 20–24-nucleotide-long non-coding RNAs that regulate gene expression by binding to the 3′ untranslated regions (3′ UTRs) of target mRNAs, thereby influencing various biological processes such as immunity and apoptosis^15^. For example, Mtb upregulates miR-33 to suppress the expression of autophagy-related molecules ATG5 and ATG12^16^, and induces miR-144* to inhibit DRAM2, thereby further suppressing autophagy^17^.

Moreover, the differential expression of specific miRNAs has been proposed as potential diagnostic and therapeutic biomarkers for TB^18,19^. For instance, Junseong et al. demonstrated that miR-199a-3p and miR-6886-3p can effectively distinguish ATB from LTBI^20^, while miR-155 has also been reported as a marker of active disease^21^.

Despite these advances, the precise mechanisms by which miRNAs regulate host responses to Mtb and their diagnostic potential remain underexplored. To address this gap, we conducted an integrated analysis combining high-throughput miRNA sequencing and machine learning approaches to identify novel miRNA biomarkers for active TB. In particular, we focused on miR-3607-3p, which was consistently identified through differential screening and machine learning algorithms, suggesting a potentially significant role in TB immunoregulation.

## Methods

### Collection and processing of expression data

**Fig. 1:**
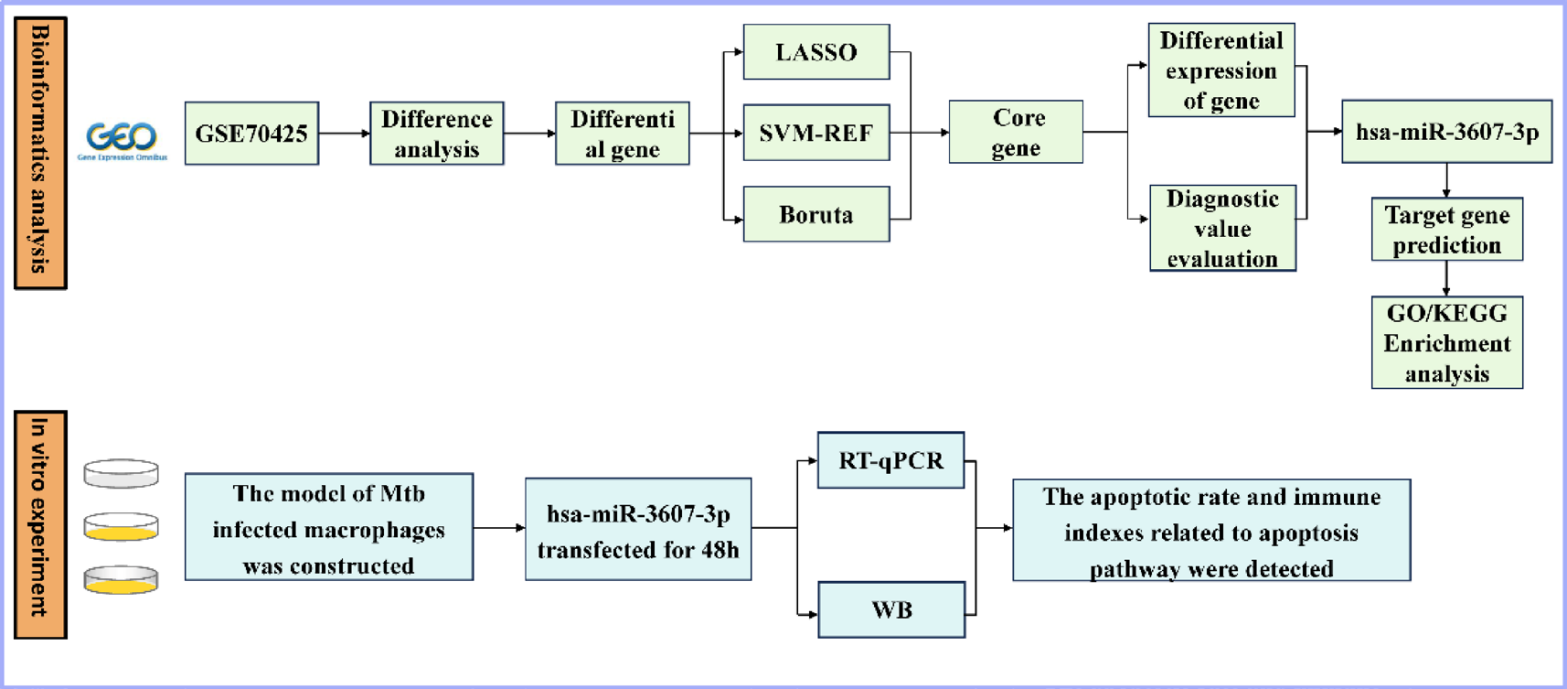
Experimental flow.

The overall workflow of the study is illustrated in Fig. 1. miRNA expression profiles associated with Mtb infection were retrieved from the GEO database (accession number: GSE70425). The selected dataset comprises 32 samples obtained from eight patients with ATB and eight individuals with LTBI, with each subject providing two independent biological replicates. For specific and detailed information, please refer to Supplementary Table 1. Data were generated using the Agilent-031181 Unrestricted_Human_miRNA_V16.0_Microarray platform.

To ensure robustness and reproducibility of the classification analysis, miRNA expression levels were normalized to reads per million (RPM) and subsequently log_2_-transformed. This preprocessing step reduces technical variability and enhances data consistency and cross-sample comparability.

### Differential expression analysis of MiRNAs

To investigate the differences in miRNA expression levels between ATB and LTBI patients, differential expression analysis was performed using the “limma” package in R. The criteria for selecting significant miRNAs were a *p*-value threshold of < 0.05 for statistical significance and |logFC| > 0.25 to highlight miRNAs exhibiting substantial expression differences. These cutoffs were based on widely accepted thresholds in miRNA research to ensure a balance between specificity and sensitivity. Differentially expressed miRNAs were visualized using the ggplot2 and pheatmap R packages, which generated volcano plots and heatmaps to clearly present the distribution and expression patterns of the significant miRNAs.

### Selection of MiRNAs using machine learning

A total of 72 differentially expressed miRNAs were initially identified. To further select key feature variables, feature selection was embedded within the 10-fold cross-validation framework to prevent data leakage. Three widely used machine learning–based feature selection algorithms were employed: LASSO regression, Support Vector Machine Recursive Feature Elimination (SVM-RFE), and the Boruta algorithm based on Random Forests.

In each fold of the cross-validation, feature selection was performed exclusively on the training subset. LASSO regression penalizes regression coefficients to retain only the most relevant variables; SVM-RFE ranks features based on their contribution to model performance and recursively eliminates less informative ones; and the Boruta algorithm identifies features that significantly contribute to classification while effectively handling multicollinearity among variables.

The intersection of the features selected by the three methods in each fold was used to construct the classification model, which was then evaluated on the corresponding validation set. Venn diagrams were used to visualize the overlap of selected features, thereby enhancing the robustness and interpretability of the selection process.

### Construction of machine learning models

Diagnostic models were developed using the intersecting miRNAs and implemented through nine different machine learning algorithms: XGBoost, SVM, Random Forest, AdaBoost, LogitBoost, Partitioning Around Medoids (PAM), Naive Bayes, Neural Network, and Bagged CART. The specific parameters of the model are shown in Supplementary Table 2. These algorithms provide diverse classification frameworks that allow for a thorough evaluation of the diagnostic value of miRNAs for active TB. All models were constructed in R version 4.3.1, and their performance was assessed through 10-fold cross-validation. The data were randomly split into training and testing sets at a ratio of 8:2. To evaluate the diagnostic capability, several metrics were employed, including the area under the receiver operating characteristic curve (AUROC), accuracy, sensitivity, specificity, positive predictive value (PPV), and negative predictive value (NPV). These metrics were used to assess both the classification accuracy and prediction stability of the models.

### Prediction of hsa-miR-3607-3p target genes

To explore the molecular regulatory mechanisms of the differentially expressed miRNAs, potential target genes were predicted using the TargetScan database. A miRNA-mRNA regulatory network was constructed and visualized with Cytoscape, which helped illustrate the regulatory interactions between miRNAs and their target genes. GO functional enrichment and KEGG pathway analyses were conducted with the “clusterProfiler” and “enrichplot” R packages, facilitating the identification of biological functions and signaling pathways associated with the target genes. Key biological processes and significantly enriched pathways (*p* < 0.05, *q* < 0.05) were identified.

### Enrichment analysis of target genes

To further understand the biological roles of the target genes of differentially expressed miRNAs, GO functional enrichment and KEGG pathway analyses were performed using the “clusterProfiler” and “enrichplot” R packages. The significance thresholds were set at *p*-value < 0.05 and *q*-value < 0.05, with the q-value adjusted for multiple hypothesis testing to control for false positives. This enrichment analysis allowed for the identification of key functional roles of the target genes in TB pathogenesis and progression.

### Bacterial culture

The *Mycobacterium tuberculosis* H37Rv strain (originally preserved in our laboratory) was cultured on Lowenstein-Jensen medium at 37 °C for 3–4 weeks, until it reached the logarithmic growth phase. Afterward, bacterial clumps were washed with sterile PBS and transferred to a sterile 50 mL centrifuge tube. The samples were centrifuged at 3000 g for 5 min, repeated twice, and resuspended in PBS. The bacterial concentration was adjusted to 1 × 10^7^ CFU/mL and stored for future experiments. The H37Rv strain was chosen as the experimental model due to its role as the standard laboratory strain of M. tuberculosis, ensuring reliable and reproducible results. The bacterial purity was verified by microscopic examination to confirm the absence of contamination.

### Cell culture

Human THP-1 macrophages (obtained from our laboratory cell bank) were cultured in RPMI 1640 medium, supplemented with 10% fetal bovine serum (FBS; PAN Biotech, Aidenbach, Germany). The cells were incubated at 37 °C in a 5% CO_2_ atmosphere until they were ready for further experimentation.

### Establishment of an Mtb-infected macrophage model

THP-1 cells (obtained from our laboratory cell bank) were cultured to the logarithmic growth phase and seeded into 24-well plates (Corning, USA) at a density of 2 × 10^5^ cells per well. The cells were treated with phorbol 12-myristate 13-acetate (PMA, final concentration: 100 ng/mL) for 24 h, followed by culture in PMA-free complete medium for an additional 48 h to induce differentiation into macrophages. After differentiation, the macrophages were infected with *Mycobacterium tuberculosis* (Mtb) at a multiplicity of infection (MOI) of 10 for 24 h. Post-infection, cell morphology was observed using light microscopy. Cell viability was assessed using the Trypan Blue exclusion assay to ensure cell survival for subsequent experiments.

### Transfection of MiRNA mimics

miR-3607-3p mimics and their negative control (NC) (GenePharma, Shanghai, China) were transfected into differentiated THP-1 macrophages using Lipofectamine 8000 transfection reagent (Beyotime, China). For transfection, the miRNA mimics and Lipofectamine 8000 were mixed at a 1:1 ratio, resulting in a final concentration of 100 nM. Following a 15-minute incubation at room temperature in serum-free medium, the mixture was added to the cells. Twenty-four hours post-transfection, total RNA was extracted, and miRNA expression levels were quantified via qRT-PCR to assess transfection efficiency.

### Colony-forming unit (CFU) assay

At 5 h post-infection, macrophages were washed three times with PBS to remove extracellular Mtb completely. The cells were then lysed using a 0.1% Triton X-100 solution to release intracellular bacteria. The resulting lysates were serially diluted tenfold and plated on Lowenstein–Jensen medium. The plates were incubated at 37 °C for three weeks, after which colony numbers were counted and expressed as CFU/mL to evaluate intracellular Mtb survival.

### Flow cytometry

The apoptosis detection kit (Solibao, Beijing, China) was used to assess apoptosis rates in different experimental groups. Experimental groups were treated according to the protocol, and apoptosis rates were determined following the manufacturer’s instructions. Furthermore, when cells reach late-stage apoptosis, they are considered dead; however, PI staining struggles to distinguish between apoptotic and necrotic cell death. Consequently, the proportion of late apoptotic and necrotic cells increases, which accounts for the observed variability.

### Western blotting

Total protein was extracted from cells and quantified using a protein assay kit. Proteins were separated by SDS-PAGE and transferred to PVDF membranes. To ensure clarity and specificity of the target protein bands, gels were pre-trimmed based on molecular weight markers to retain only the region containing the protein of interest. The trimmed gels were transferred to PVDF membranes and incubated overnight with appropriate primary antibodies at 4 °C. The following day, secondary antibodies were applied, and an enhanced chemiluminescent reagent (Solarbio, Beijing, China) was used to detect the antigen-antibody complexes. Gray value analysis was performed using ImageJ software (ImageJ v2.0.0, US National Institutes of Health).

### Quantitative real-time PCR

Total RNA was extracted from THP-1-derived macrophages using a commercial kit (TIANGEN Biotech, Beijing, China), followed by reverse transcription to synthesize cDNA. qPCR using SYBR Green dye was performed on an Applied Biosystems real-time PCR system (USA), with reaction components prepared according to the manufacturer’s protocol. For the analysis of clinical samples, total RNA was extracted from the peripheral blood of five patients with ATB and five individuals with LTBI. miRNA-specific reverse transcription followed by qPCR was performed using a miRNA quantification kit. Relative expression levels were calculated using the 2^− ΔΔCt^ method, with U6 serving as the internal control. Primer sequences are listed in Table 1.

**Table 1:** Primers sequence.

Gene	Primer (5′–3′)	
β-actin	F: GGCTGTATTCCCCTCCATCG	R: CCAGTTGGTAACAATGCCATGT
Caspase-3	F: ATGGAGAACAACAAAACCTCAGT	R: TTGCTCCCATGTATGGTCTTTAC
Caspase-8	F: TGCTTGGACTACATCCCACAC	R: TGCAGTCTAGGAAGTTGACCA
Caspase-9	F: TCCTGGTACATCGAGACCTTG	R: AAGTCCCTTTCGCAGAAACAG
PARP	F: TCCTGGTACATCGAGACCTTG	R: AAGTCCCTTTCGCAGAAACAG
hsa-miR-3607-3p	F: ATGACTGTAAACGCTTTCTG	R: GTGCAGGGGTCCGAGGT
U6	F: TGCGGGTGCTCGCTTCGGCAGC	R: GTGCAGGGTCCGAGGT

### Statistical analysis

For comparisons between two groups, the Wilcoxon rank-sum test was used to analyze relationships between continuous variables. For comparisons involving three or more groups, one-way ANOVA was applied to assess differences between groups, followed by Tukey’s post-hoc test for significant results. Data normality was evaluated using the Shapiro-Wilk test, and homogeneity of variances was assessed with Levene’s test. A *P*-value < 0.05 was considered statistically significant. All statistical analyses were performed using R software (version 4.3.1) and Prism 9 (GraphPad Software, USA). Detailed statistical results, including *P*-values and effect sizes (e.g., Cohen’s d), are provided in the supplementary materials to enhance transparency and reproducibility.

## Results

### Differential expression analysis

**Fig. 2:**
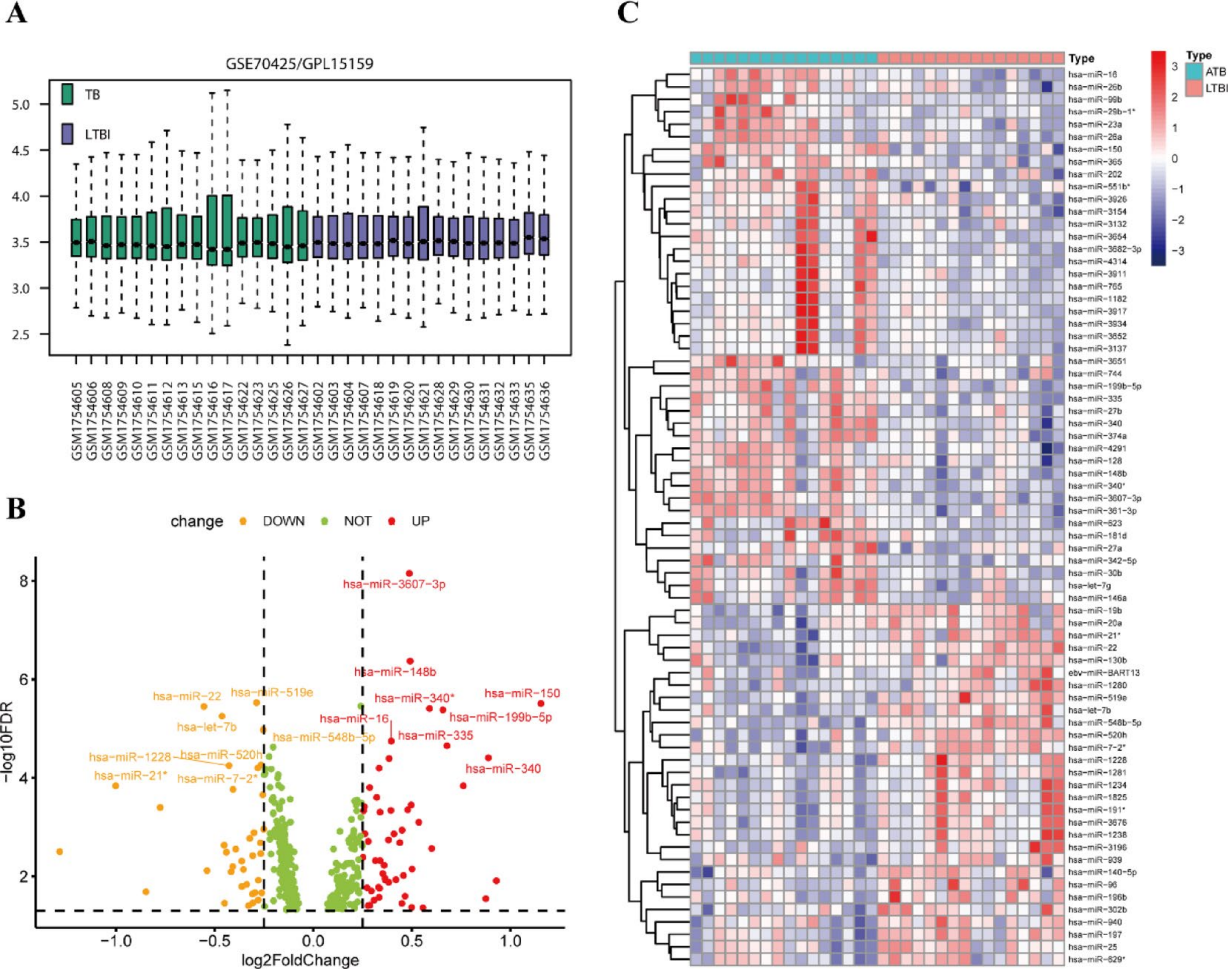
Differential Expression Analysis. (**A**) Normalization of the GSE70425 dataset. Boxplot of miRNA expression levels for samples from the GSE70425/GPL15159 dataset, comparing samples from individuals with tuberculosis (TB, n = 16) and latent tuberculosis infection (LTBI, n = 16). (**B**) Volcano plot of differentially expressed miRNAs between TB and LTBI samples. The x-axis represents the log2 fold change (log2FC), and the y-axis represents the − log10 false discovery rate (FDR). (**C**) Heatmap of miRNA expression levels in TB and LTBI samples. The expression of each miRNA is shown across different samples.

To identify potential miRNA biomarkers that differentiate ATB from LTBI, the miRNA dataset GSE70425 was retrieved from the GEO database. The dataset underwent log2 transformation and normalization to correct for technical biases and ensure data consistency. The normalization results revealed that the average expression values for all samples were approximately 3.5, indicating high-quality data (Fig. 2A). Using stringent criteria of *p*-value < 0.05 and False Discovery Rate (FDR) < 0.05, 72 miRNAs were found to be differentially expressed. Among these, 43 miRNAs were significantly upregulated in ATB samples, while 29 were significantly downregulated in LTBI samples. Volcano plot analysis highlighted hsa-miR-3607-3p as notably upregulated in ATB patients (Fig. 2B). Heatmap visualization further confirmed the elevated expression of hsa-miR-3607-3p in ATB samples, showing distinct group specificity (Fig. 2C). These findings suggest that hsa-miR-3607-3p may play a pivotal role in the pathogenesis of tuberculosis.

### Machine learning-based selection of feature MiRNAs

**Fig. 3:**
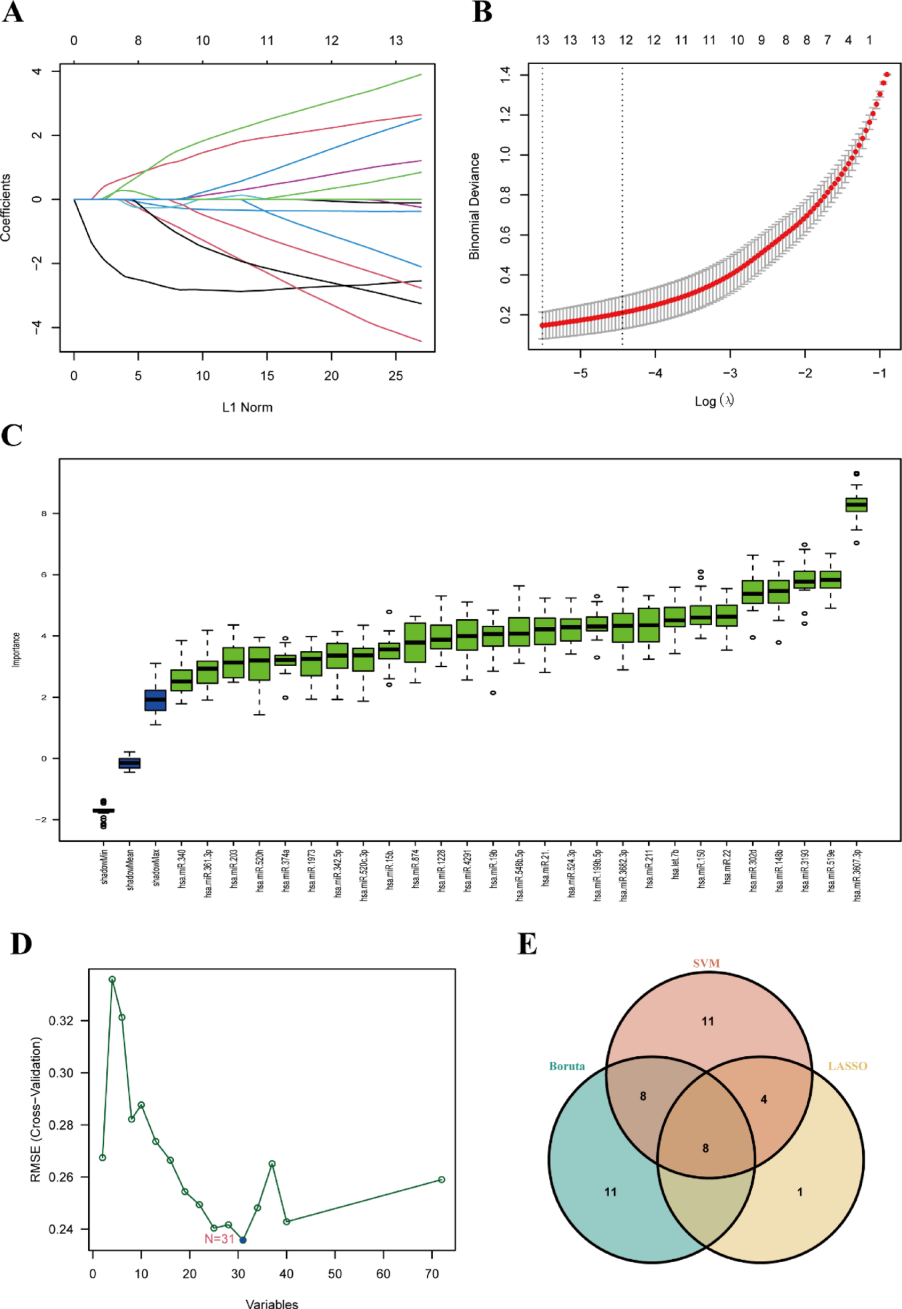
Feature miRNA selection. (**A**) Lasso regression coefficient plot, showing the change in coefficients of selected variables as the L1 regularization parameter (λ) increases. The coefficients are represented by colored lines, with each line corresponding to a specific variable. (**B**) Binomial deviance plot showing the performance of the model as a function of the log-transformed λ values. The red line indicates the optimal λ value selected by cross-validation, where the deviance is minimized. (**C**) Feature miRNA selection using the Boruta algorithm.Boxplot showing the distribution of response variables across different selected features. The boxplot summarizes the minimum, first quartile, median, third quartile, and maximum values for each feature. (**D**) Feature miRNA selection using SVM.Cross-validation RMSE plot against the number of variables. The plot shows the RMSE decreasing as the number of variables increases, with the optimal number of variables selected based on the lowest RMSE. (**E**) Venn diagram illustrating the overlap of variables selected by three different feature selection methods: Support Vector Machine (SVM), Boruta, and Lasso. The numbers indicate the count of variables selected by each method and their intersection.

To refine the identification of potential miRNA biomarkers for ATB, three machine learning methods were applied: LASSO regression, the Boruta algorithm and SVM-RFE. LASSO regression, which performs both variable selection and regularization in high-dimensional datasets, identified 13 candidate miRNAs (Fig. 3A,B). The Boruta algorithm, based on the Random Forest model, identified 27 key miRNAs as the most significant variables (Fig. 3C). SVM-RFE ranked the importance of feature variables, resulting in the identification of 31 candidate miRNAs (Fig. 3D). A Venn diagram was used to display the overlap of miRNAs identified by all three methods, revealing a set of eight robust miRNAs, including hsa-miR-3607-3p, hsa-miR-148b, hsa-miR-519e, hsa-miR-150, hsa-miR-199b-5p, hsa-let-7b, hsa-miR-548b-5p, and hsa-miR-19b (Fig. 3E). The intersection of these miRNAs reduced the likelihood of false-positive results, enhancing the stability and reliability of the identified biomarkers, which could have significant clinical value in diagnosing both ATB and LTBI.

### Selection and diagnostic performance of target MiRNAs

**Fig. 4:**
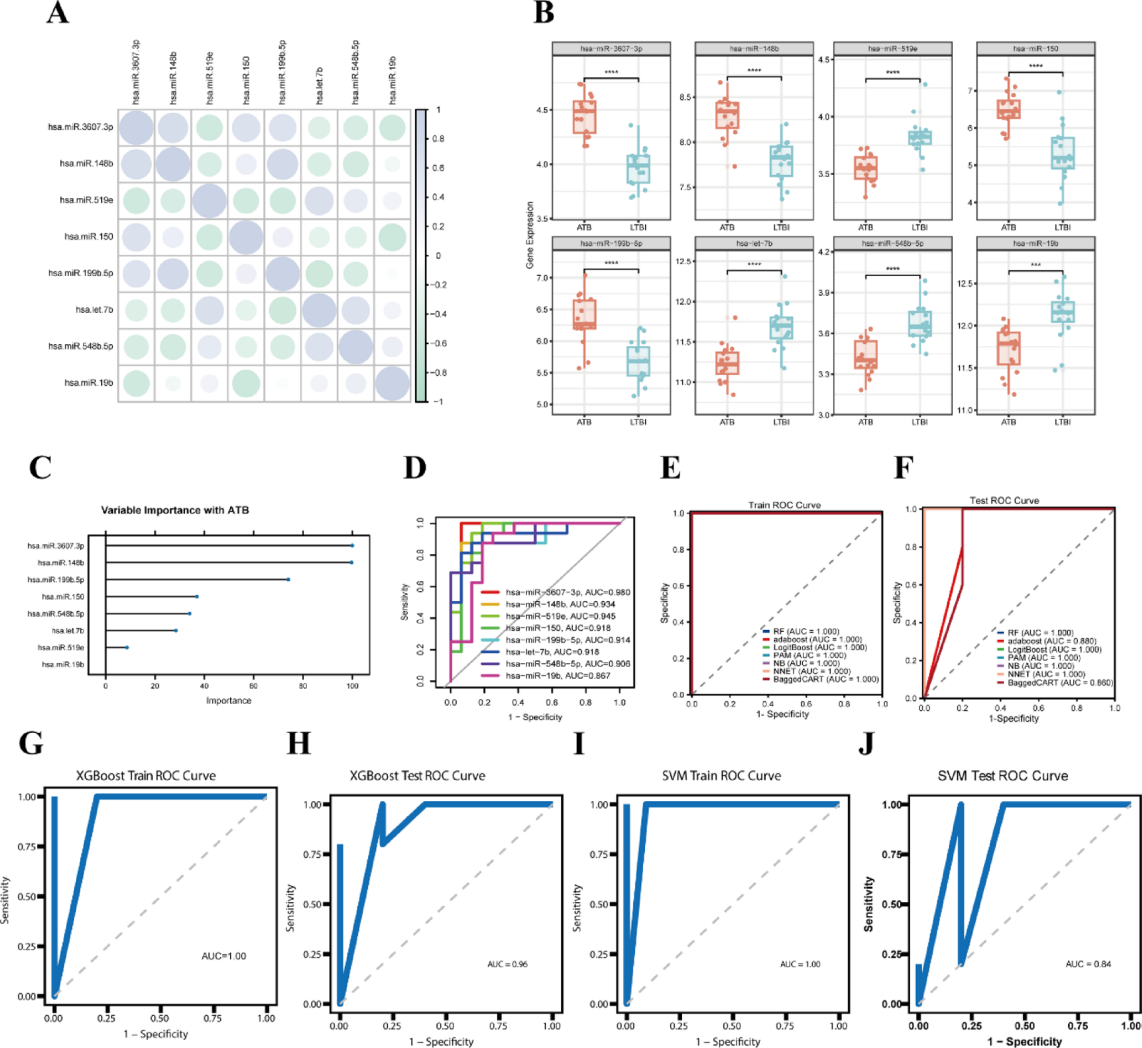
Machine learning-based predictive model construction. (**A**) Correlation matrix of miRNA expressions, showing the correlation between different miRNAs. Each circle represents the correlation between two miRNAs, with the size and color indicating the strength and direction of the correlation. (**B**) Box plots comparing the gene expression levels between the AtTB and LTBI groups for each miRNA. Statistically significant differences are indicated by asterisks, with **** representing *p*-values < 0.0001. (**C**) Variable importance plot showing the importance of selected miRNAs in predicting TB. The length of the bars indicates the relative importance of each miRNA. (**D**) ROC curve for the classification performance of various models. Each curve represents the trade-off between sensitivity and 1-specificity for the selected model. (**E**) ROC curve for the training dataset, showing the performance of different models. The AUC values for each model are listed in the legend. (**F**) ROC curve for the test dataset, demonstrating the performance of various models on unseen data. The AUC values are also provided for each model. (**G**) XGBoost training dataset ROC curve. (**H**) XGBoost test dataset ROC curve. (**I**) SVM training dataset ROC curve. (**J**) SVM test dataset ROC curve.

To evaluate the diagnostic potential of the identified miRNAs, we conducted a comprehensive analysis of their expression levels and clinical relevance. Our results revealed that hsa-miR-3607-3p, hsa-miR-148b, hsa-miR-150, and hsa-miR-199b-5p were significantly upregulated in patients with ATB, while hsa-miR-519e, hsa-let-7b, hsa-miR-548b-5p, and hsa-miR-19b showed higher expression in individuals with LTBI (Fig. 4A,B). Further analysis using SHAP values and feature importance demonstrated that hsa-miR-3607-3p played a pivotal role in the classification model (Figs. 4C and SF1). ROC curve analysis revealed that hsa-miR-3607-3p alone achieved an AUC of 0.980 in distinguishing between ATB and LTBI (Fig. 4D), indicating its exceptional diagnostic performance. Consequently, hsa-miR-3607-3p was identified as the key candidate miRNA in this study. Additionally, we validated the expression of hsa-miR-3607-3p in clinical samples, confirming its significant upregulation in ATB patients (Fig. SF2). This result is consistent with the bioinformatics findings, further supporting its reliability as a potential biomarker.

Despite the promising diagnostic potential of individual miRNAs, we further integrated all nine miRNAs to construct a suite of machine learning models, including XGBoost, SVM, Random Forest, AdaBoost, LogitBoost, PAM, Naive Bayes, neural network classifiers, and Bagged CART, aiming to augment predictive performance. In the training cohort, all models exhibited outstanding classification capability, with AUC values reaching 1.000 (Fig. 4E,J). In the independent test cohort, XGBoost (AUC = 0.96), SVM (AUC = 0.84), AdaBoost (AUC = 0.88), and Bagged CART (AUC = 0.86) consistently maintained high diagnostic accuracy.

To address the inherent class imbalance between ATB and LTBI samples, automatic class-weight adjustment mechanisms were implemented in models such as SVM and XGBoost, effectively mitigating bias toward the majority class. Additionally, we conducted a comprehensive assessment of model generalizability by incorporating F1-score, Precision, and Recall, along with Precision–Recall (PR) curves, to further evaluate model robustness under imbalanced data conditions (Supplementary Table 3). All nine models exhibited favorable performance across these metrics.

In summary, whether utilized independently (as with hsa-miR-3607-3p) or as a combinatorial miRNA panel, the selected miRNAs demonstrated exceptional diagnostic efficacy in differentiating ATB from LTBI, thereby providing compelling molecular evidence to substantiate their clinical utility in early tuberculosis diagnosis.

### Target gene prediction and functional enrichment analysis of hsa-miR-3607-3p

**Fig. 5:**
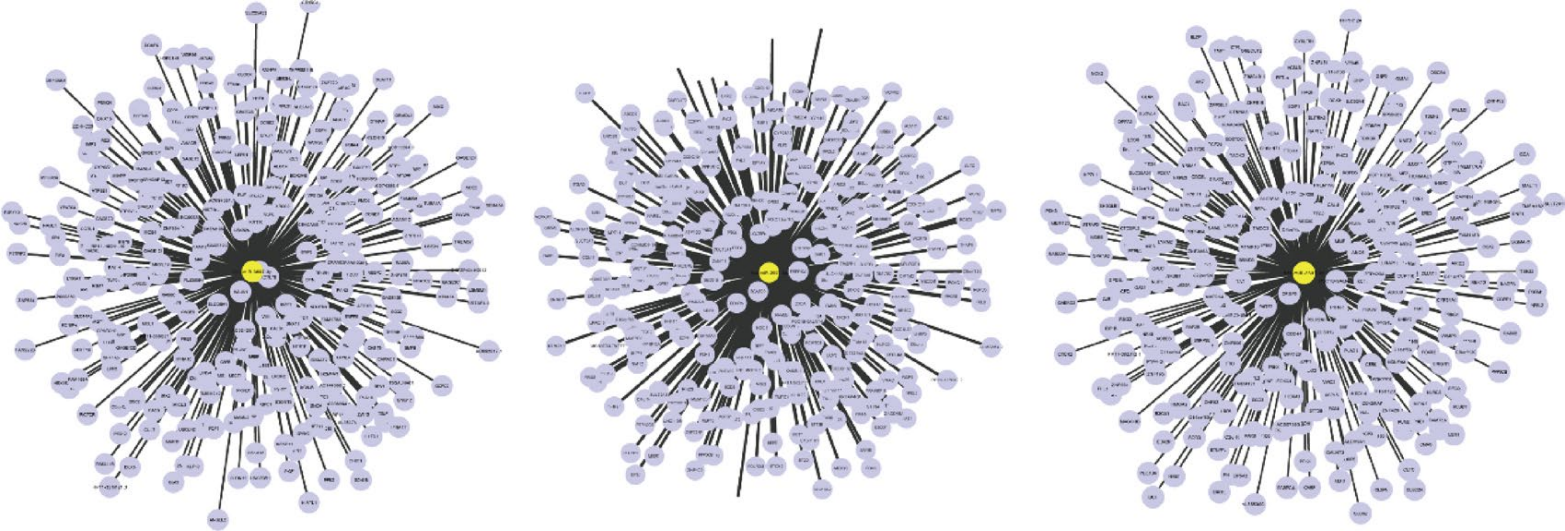
Construction of the miRNA-mRNA axis.

To further investigate the potential molecular mechanism of hsa-miR-3607-3p in ATB, the TargetScan and miRDB databases were utilized to predict its target genes, applying stringent screening criteria of *P* < 0.05 and a total TargetScan score exceeding 90. By integrating the results from these two databases, a set of pivotal target genes potentially modulated by hsa-miR-3607-3p was selected, and Cytoscape software was employed to construct the miRNA-mRNA regulatory network (Fig. 5). This network implies that hsa-miR-3607-3p may exert influence over a variety of biological processes through the modulation of its target genes.

**Fig. 6:**
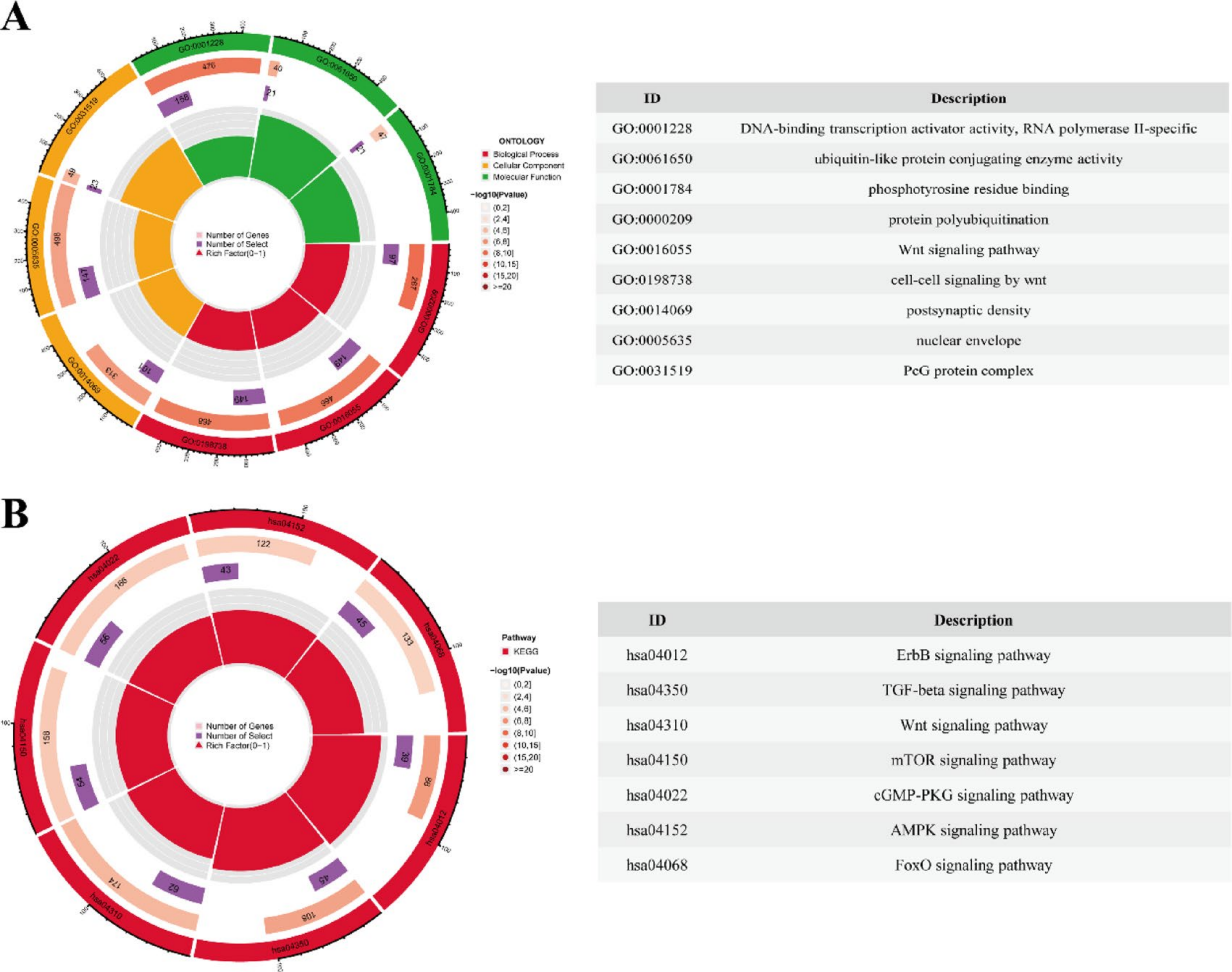
Functional enrichment analysis. (**A**) Gene Ontology (GO) enrichment analysis of the target genes of hsa-miR-3607-3p. The circular plot shows the number of genes (represented by the inner circle) and the number of selected genes (represented by the outer circle) in different GO categories. The colors indicate the categories: Biological Process (red), Cellular Component (orange), and Molecular Function (green). The size of each segment represents the number of genes involved in each category, and the intensity of the color corresponds to the − log10(*p*-value), with darker shades indicating more significant enrichment. (**B**) Kyoto Encyclopedia of Genes and Genomes (KEGG) pathway enrichment analysis of the target genes of hsa-miR-3607-3p. The circular plot shows the number of genes (represented by the inner circle) and the number of selected genes (represented by the outer circle) in different KEGG pathways. The colors represent the significance of each pathway: with red indicating more significant pathways. The pathway names are listed, and the size of each segment corresponds to the number of genes associated with each pathway. The intensity of the color represents the − log10(*p*-value), with darker shades indicating more significant enrichment.The pathways were generated using KEGG Mapper (https://www.kegg.jp/kegg/tool/map_pathway.html) and are reproduced with permission from Kanehisa Laboratories ©2025.

Subsequently, GO and KEGG enrichment analyses were conducted to further elucidate the functional attributes of the identified target genes. GO analysis revealed that these target genes were predominantly localized in cellular compartments such as the nucleus and cytoplasm, involved in critical biological processes including protein ubiquitination and intracellular signaling, and were significantly enriched in molecular functions such as RNA binding, protein binding, and DNA binding (Fig. 6A). KEGG pathway analysisidentified significant enrichment in pathways associated with mTOR signaling, AMP*K* signaling, FoxO signaling, and other inflammation- and apoptosis-related pathways (Fig. 6B)^22,23^. These pathways indicate that hsa-miR-3607-3p may regulate ATB through the modulation of apoptotic and inflammatory processes.

### Effects of hsa-miR-3607-3p on Mtb-Infected THP-1 macrophages

**Fig. 7:**
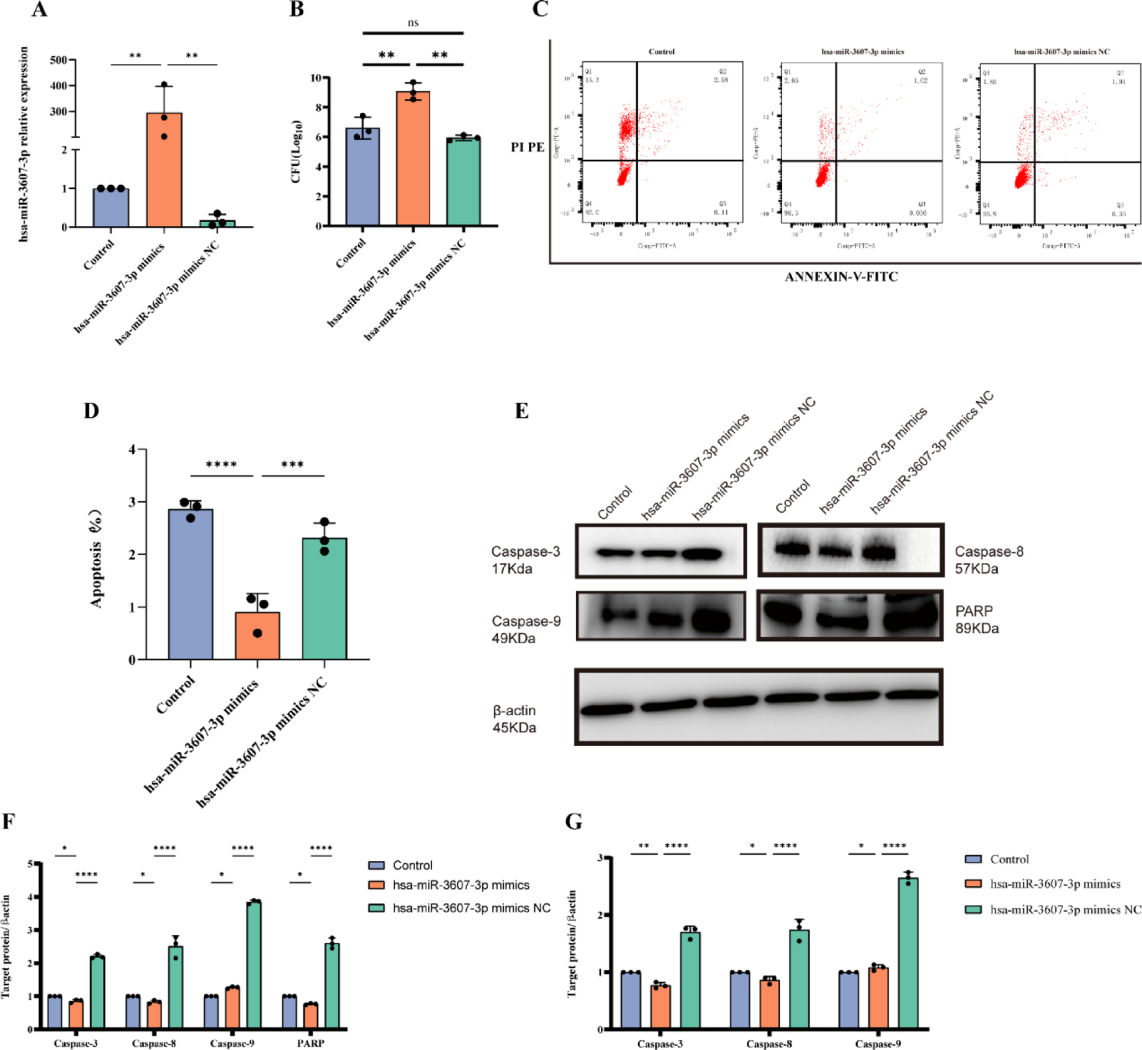
Mechanistic investigation of miR-3607-3p in regulating apoptosis in THP-1-derived macrophages. (**A**) qRT-PCR analysis of miR-3607-3p expression 24 h post-transfection, shown as fold change relative to the control group (n = 3 biological replicates, 3 independent experiments, analyzed by two-tailed *t*-test comparing control vs. hsa-miR-3607-3p mimics group). (**B**) Colony-forming unit (CFU) count in macrophages infected with *Mycobacterium tuberculosis* (Mtb), comparing control, hsa-miR-3607-3p mimics, and hsa-miR-3607-3p mimics NC groups (n = 3 biological replicates, 3 independent experiments, analyzed by one-way ANOVA, no significant difference observed, ns: not significant). (**C**) Flow cytometry analysis of apoptosis in control, hsa-miR-3607-3p mimics, and hsa-miR-3607-3p mimics NC groups, with the upper right quadrant representing apoptotic cells marked by Annexin-V-FITC and PI staining (n = 3 biological replicates, 3 independent experiments). (**D**) Quantification of apoptosis levels in Mtb-infected THP-1-derived macrophages, showing significantly reduced apoptosis in the hsa-miR-3607-3p mimics group compared to control and NC mimics groups (n = 3 biological replicates, 3 independent experiments, analyzed by one-way ANOVA, *P < 0.05). (**E**) Western blot analysis of apoptosis-related proteins (Caspase-3, Caspase-8, Caspase-9, and PARP) in different groups, with β-actin as the loading control (n = 3 biological replicates, 3 independent experiments). (**F**) Quantification of apoptosis-related protein expression (Caspase-3, Caspase-8, Caspase-9, and PARP), showing significantly increased expression of Caspase-3, Caspase-9, and PARP in the hsa-miR-3607-3p mimics group compared to control and NC mimics groups (n = 3 biological replicates, 3 independent experiments, analyzed by one-way ANOVA, **P < 0.01, ***P < 0.001). (**G**) qRT-PCR analysis of apoptosis-related gene expression, showing significant differences in Caspase-3, Caspase-8, and Caspase-9 levels between groups (n = 3 biological replicates, 3 independent experiments, analyzed by one-way ANOVA, *P < 0.05, **P < 0.01, ****P < 0.0001). Group Definitions: The control group refers to untreated cells; the hsa-miR-3607-3p mimics group refers to Mtb-infected macrophages transfected with miR-3607-3p mimics; the hsa-miR-3607-3p mimics NC group refers to Mtb-infected macrophages transfected with a scrambled negative control mimic. Detailed statistical analyses and raw data are provided in the supplementary materials.

To elucidate the functional role of hsa-miR-3607-3p in Mtb infection, hsa-miR-3607-3p mimics were transfected into THP-1 macrophages to investigate its influence on host–pathogen dynamics. Analysis of transfection efficiency revealed a markedly elevated expression of hsa-miR-3607-3p in the mimics group compared to both the control and negative control cohorts (*P* < 0.05, Fig. 7A), thereby validating the reliability of downstream experimental findings.

A colony-forming unit (CFU) assay demonstrated that the intracellular persistence of Mtb was markedly enhanced following hsa-miR-3607-3p transfection, exhibiting statistically significant differences relative to both the control and negative control groups (*P* < 0.05, Fig. 7B). These observations imply that hsa-miR-3607-3p may facilitate Mtb survival within macrophages by attenuating the host’s antimicrobial defense mechanisms.

Moreover, flow cytometric analysis revealed that hsa-miR-3607-3p significantly suppressed both early and late apoptosis in Mtb-infected THP-1 macrophages. Compared to the negative control group, the proportion of apoptotic cells was significantly diminished (*P* < 0.05, Fig. 7C,D). To probe the underlying molecular mechanism, we interrogated the expression profiles of pivotal molecules involved in the caspase-dependent apoptotic cascade at both the transcript and protein levels. The expression levels of caspase-9, caspase-8, caspase-3, and PARP were markedly downregulated in the hsa-miR-3607-3p-transfected group (*P* < 0.05, Fig. 7E,G), suggesting that hsa-miR-3607-3p may modulate Mtb-induced macrophage apoptosis via a caspase-dependent pathway.

## Discussion

Mtb infection induces a multifaceted immune response in the host, wherein the differential expression of coding and non-coding RNAs across various stages significantly modulates the host’s immune functionality. MicroRNAs, a pivotal class of non-coding RNAs, have been extensively documented for their regulatory roles in key immune processes such as autophagy, inflammation modulation, and apoptosis, primarily through post-transcriptional gene regulation^24^. Therefore, investigating the regulatory mechanisms of miRNAs in tuberculosis is of paramount significance for comprehending the pathogenesis and progression of the disease.

In this study, the GSE70425 dataset was comprehensively analyzed using a range of machine learning approaches to pinpoint potential miRNA biomarkers and therapeutic targets. These methodologies are adept at handling high-dimensional data, mitigating overfitting, and ensuring the robustness and validity of the findings. A total of eight core miRNAs were identified, with hsa-miR-3607-3p, hsa-miR-148b, hsa-miR-150, and hsa-miR-199b-5p exhibiting markedly higher expression levels in ATB samples, whereas hsa-miR-519e, hsa-let-7b, hsa-miR-548b-5p, and hsa-miR-19b showed elevated expression in LTBI samples.

To rigorously assess the clinical diagnostic potential of the identified core miRNAs, we employed receiver operating characteristic (ROC) curves and logistic regression analyses. The results revealed that all eight core miRNAs exhibited areas under the curve (AUCs) surpassing 0.8, with hsa-miR-3607-3p demonstrating exceptional diagnostic prowess, achieving an AUC of 0.980. This superior performance underscores its capability to effectively differentiate active tuberculosis (ATB) from latent tuberculosis infection (LTBI), justifying its selection as the primary target for subsequent functional validation in this study. Notably, in comparison to prior studies—such as those reporting miR-29a-3p^25^ with an AUC of 0.808 and miR-374c-5p^20^ with an AUC of 0.86 as potential TB biomarkers—hsa-miR-3607-3p in our investigation exhibited markedly superior discriminatory power, highlighting its substantial potential for clinical application.

Moreover, leveraging the expression profiles of these eight core miRNAs, we developed a sophisticated clinical diagnostic model integrating diverse machine learning methodologies. This model demonstrated remarkable precision, sensitivity, specificity, and predictive accuracy in distinguishing ATB from LTBI samples. Although the fold changes of certain miRNAs, including hsa-miR-3607-3p, were below 0.5, such subtle alterations in miRNA expression within clinical samples can still hold profound statistical and biological significance. Given the robust statistical significance, consistent model stability, and elevated AUC values, we posit that these miRNAs not only serve as promising candidate biomarkers for tuberculosis but also likely exert pivotal regulatory functions in its pathogenesis.

Building on the differential miRNAs identified in this investigation, we propose that these miRNAs play a central regulatory role in Mtb infection by modulating disease-associated molecular networks. Through the integration of bioinformatics tools and publicly available miRNA-mRNA databases, we identified potential target genes of hsa-miR-3607-3p and constructed miRNA-mRNA regulatory networks. PPI network analysis revealed a set of key target genes intricately linked to hsa-miR-3607-3p. Subsequent GO and KEGG enrichment analyses highlighted significant enrichment of these target genes in pathways related to inflammation and apoptosis. These findings suggest that hsa-miR-3607-3p orchestrates the immune response to Mtb through modulation of apoptosis, immune response, and pathogen clearance, thereby offering critical insights into its biological functions and potential as a therapeutic target.

Peripheral blood mononuclear cells (PBMCs) can rapidly mount an immune defense response, which is accompanied by significant changes in their miRNA expression profiles^24–26^. The immunoregulatory roles of miRNAs allow for the detection of subtle changes in miRNA expression, even preceding alterations at the protein level. These characteristics establish miRNAs as promising early diagnostic tools for detecting both minor and severe cellular injuries.

This study identified significant differences in the expression of miRNAs, such as hsa-miR-148b, between ATB and LTBI samples^27–30^. The roles of these miRNAs in various diseases have been established. For instance, hsa-miR-148b inhibits the proliferation of vascular smooth muscle cells by regulating heat shock protein 90 (HSP90), a mechanism closely linked to the progression of atherosclerosis. Conversely, hsa-miR-150 plays a crucial role in the development of tumors and hematologic disorders, with changes in its serum levels potentially serving as an early diagnostic marker. Additionally, other miRNAs, such as hsa-miR-519e, hsa-let-7b, hsa-miR-548b-5p, and hsa-miR-199b-5p, have been documented in the existing literature^30–34^.

Notably, our exosomal miRNA analysis revealed that hsa-miR-3607-3p was significantly enriched in extracellular vesicles (EVs) derived from immune cells, indicating its potential involvement in modulating the host’s anti-tuberculosis response. Previous studies have demonstrated that hsa-miR-3607-3p plays a pivotal role in multiple tumor types. For instance, in non-small cell lung cancer, hsa-miR-3607-3p significantly suppresses tumor cell proliferation and migration by inhibiting the PI3K/Akt signaling pathway^34^. In pancreatic cancer, miR-3607-3p directly targets IL-26, thereby inhibiting malignant transformation^35^. These findings suggest that hsa-miR-3607-3p may exert a similar regulatory function in Mtb infection by modulating immune-related signaling pathways.

To further investigate the functional role of hsa-miR-3607-3p in Mtb infection, we conducted functional validation using a THP-1 macrophage infection model. Experimental results demonstrated that overexpression of hsa-miR-3607-3p significantly downregulated the expression of caspase-9, caspase-8, caspase-3, and PARP, thereby inhibiting macrophage apoptosis post-infection. These findings are consistent with the signaling pathway trends identified by enrichment analysis, suggesting that hsa-miR-3607-3p may enhance Mtb survival within host cells by modulating the caspase-dependent apoptotic pathway.

## Conclusion

This study provides preliminary evidence supporting the critical immunoregulatory role of hsa-miR-3607-3p in Mtb infection. By downregulating the expression of Caspase-9, Caspase-8, Caspase-3, and PARP, hsa-miR-3607-3p significantly inhibits apoptosis in THP-1-derived macrophages, potentially promoting Mtb survival within host cells. This finding highlights the potential of hsa-miR-3607-3p as a biomarker and therapeutic target for active tuberculosis, offering insights for developing novel diagnostic and therapeutic strategies. However, several limitations remain. First, the study relied on a single dataset (GSE70425) and lacks validation using an independent external cohort. Future studies should incorporate datasets from diverse populations to assess the robustness and generalizability of the findings. Second, functional assays were performed exclusively in vitro using THP-1 cells, without subsequent validation in animal models or clinical specimens. Future research should further investigate its biological roles in vivo and evaluate its feasibility as a diagnostic or therapeutic target.

## Supplementary Information

Below is the link to the electronic supplementary material.

Supplementary Material 1

Supplementary Material 2

Supplementary Material 3

Supplementary Material 5

Supplementary Material 6

Supplementary Material 4

## Data Availability

The datasets generated and/or analyzed during this study are available in the GEO (Gene Expression Omnibus) repository. The direct link to access the gene expression and clinical pathology dataset is: https://www.ncbi.nlm.nih.gov/geo/query/acc.cgi?acc=GSE70425. This link provides access to the original dataset used in our analysis. The raw data supporting the findings of this study are available upon reasonable request. Researchers interested in accessing the data should contact the corresponding author, at [zihancai001@163.com]. Requests will be reviewed to ensure compliance with ethical and privacy standards.
