# Supplementary material for: Machine learning identifies MiRNA biomarkers and immune mechanisms in active tuberculosis
Source: Sci Rep. 2025 Oct 16;15:36246. doi: 10.1038/s41598-025-20112-8 (PMC12533189; doi:10.1038/s41598-025-20112-8)

Caspase-3 Repeat1

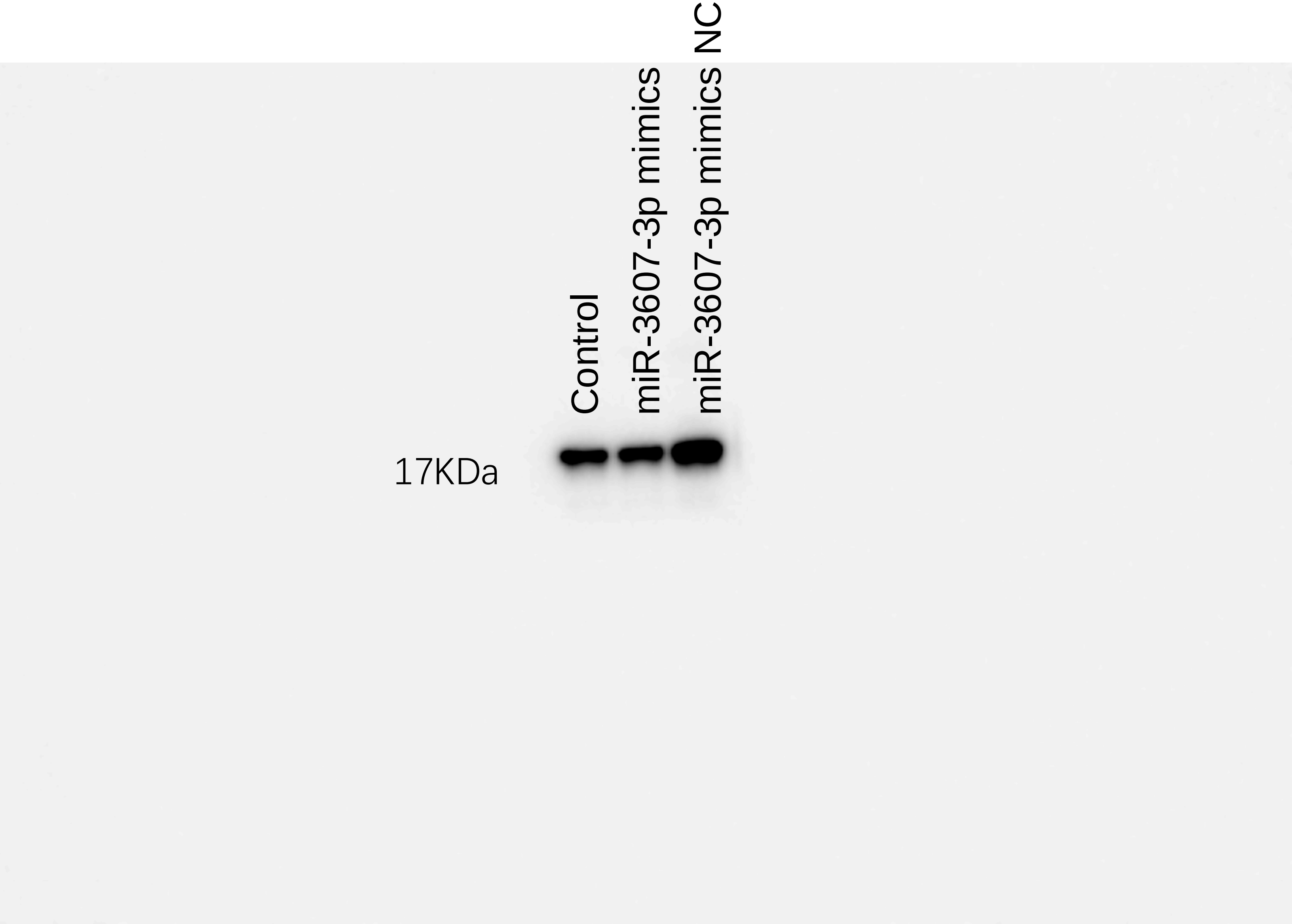

Caspase-3 Repeat2

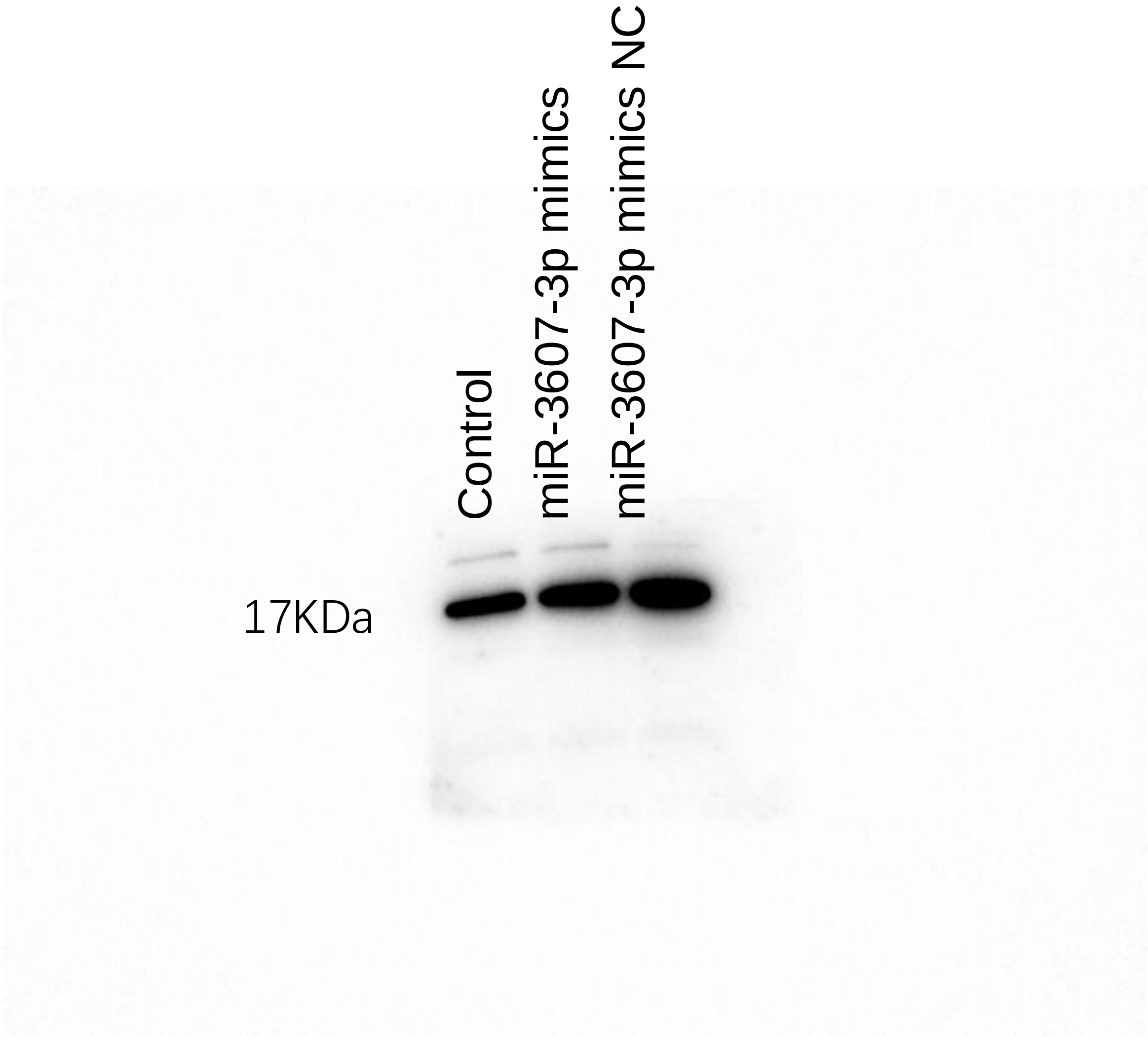

Caspase-3 Repeat3

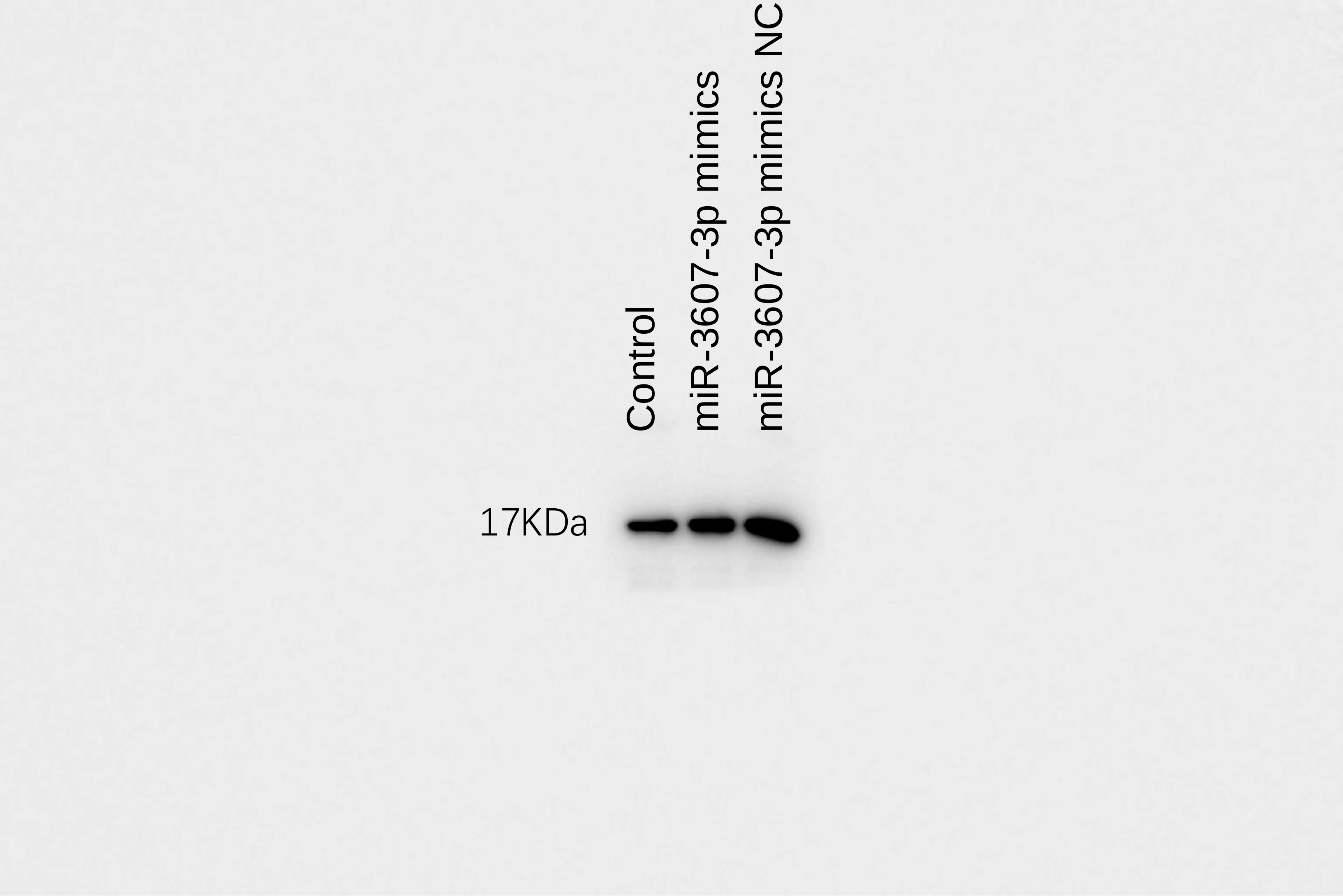

Caspase-8 Repeat1

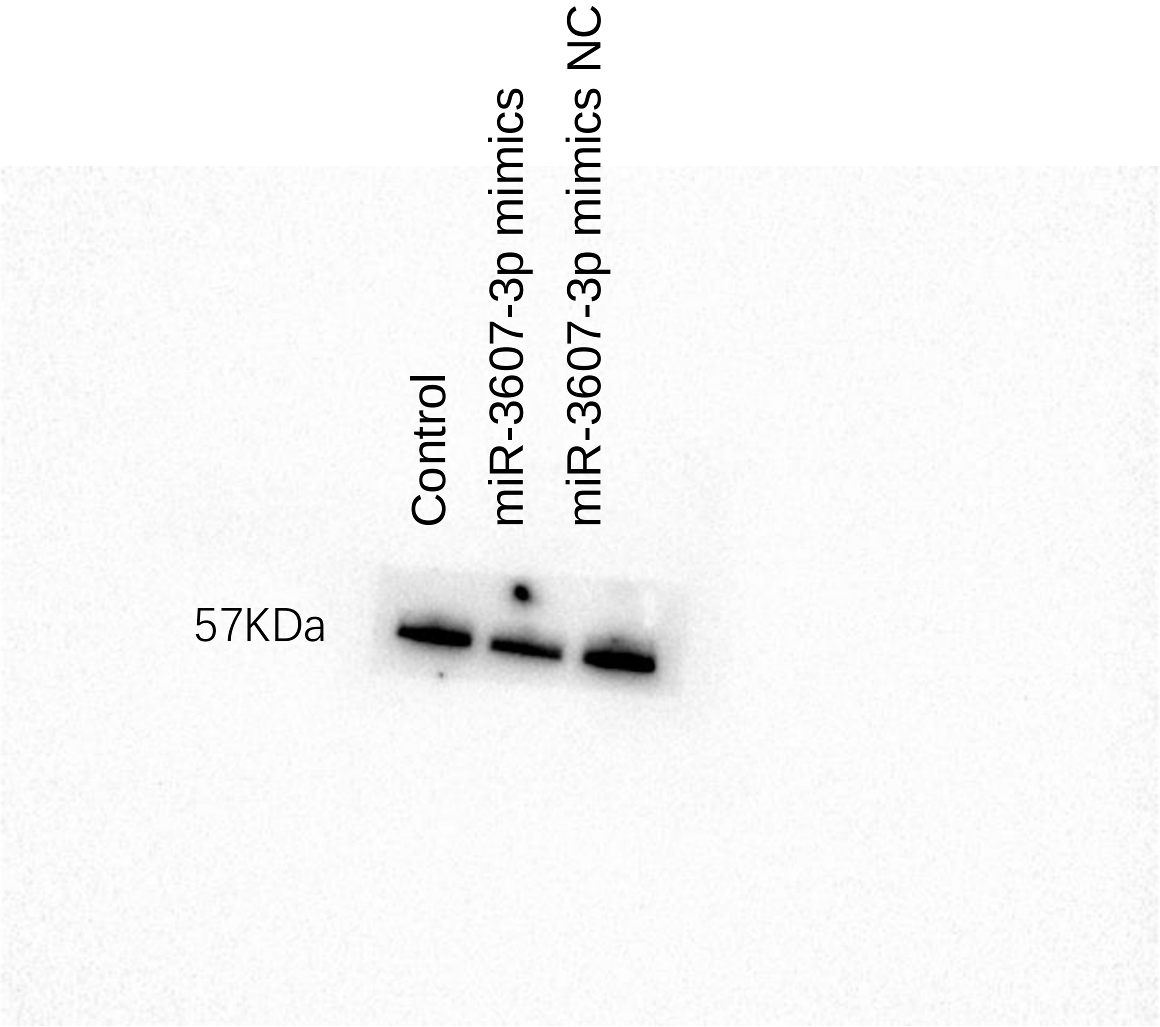

Caspase-8 Repeat2

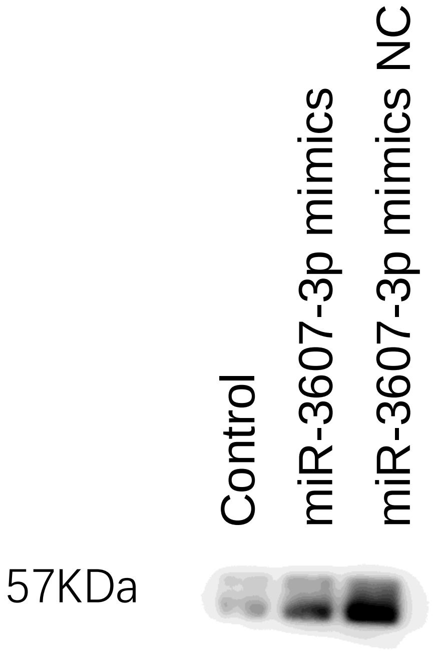

Caspase-8 Repeat3

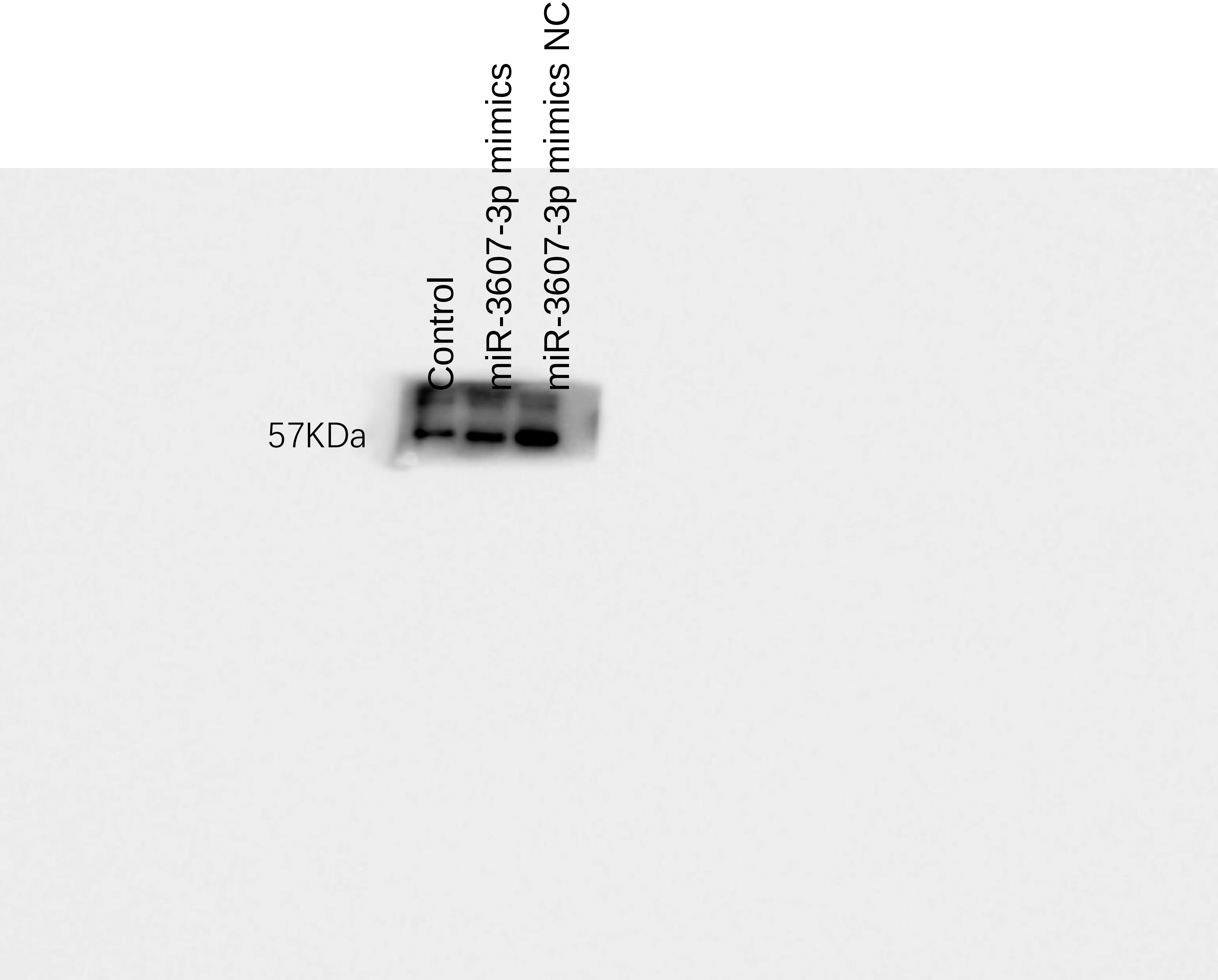

Caspase-9 Repeat1

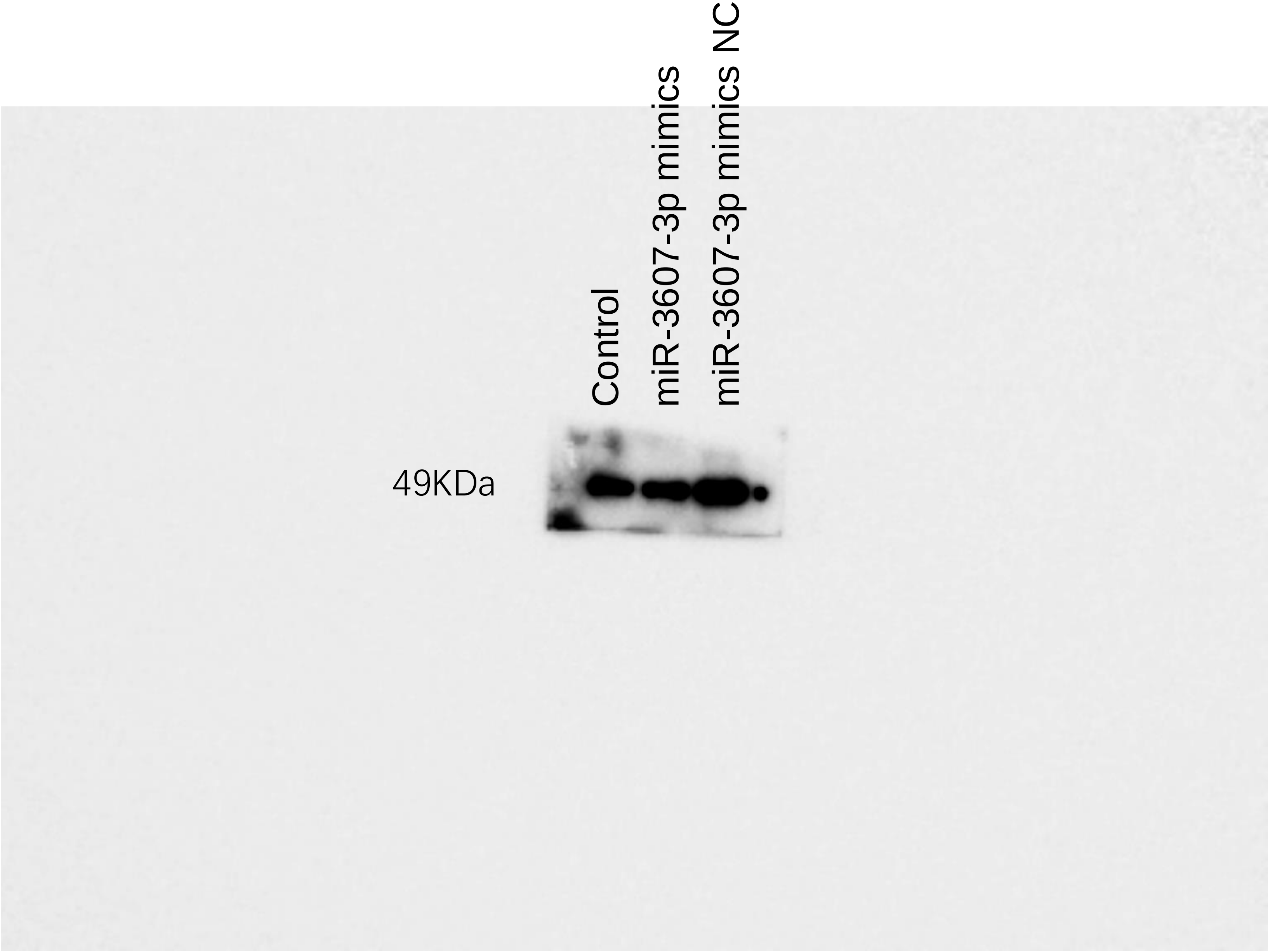

Caspase-9 Repeat2

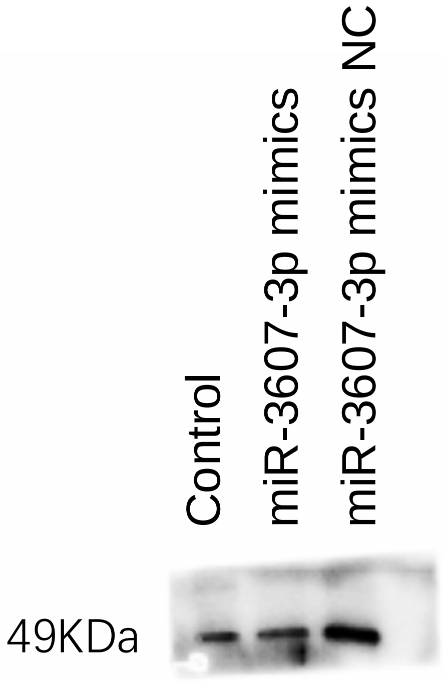

Caspase-9 Repeat3

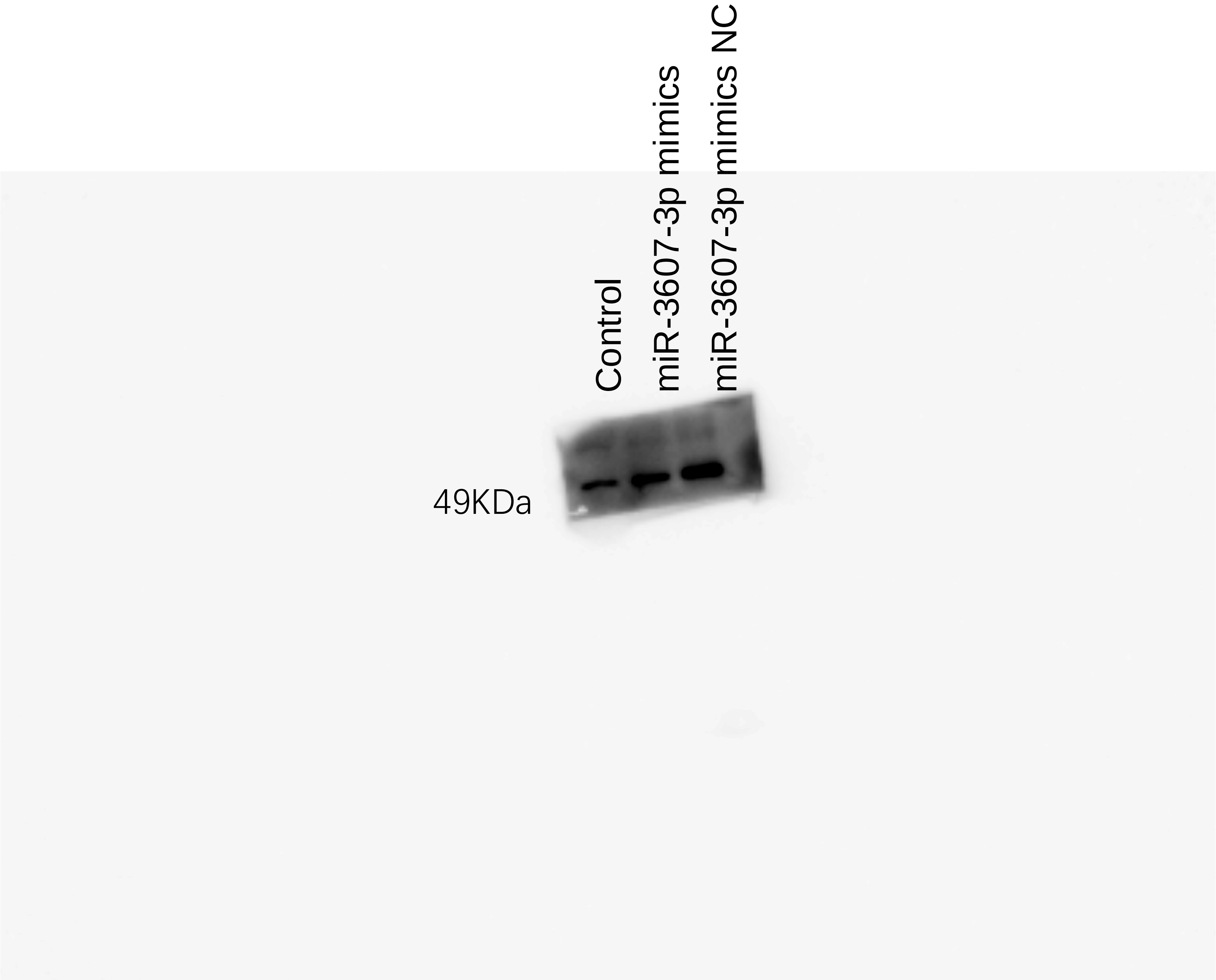

PARP Repeat1

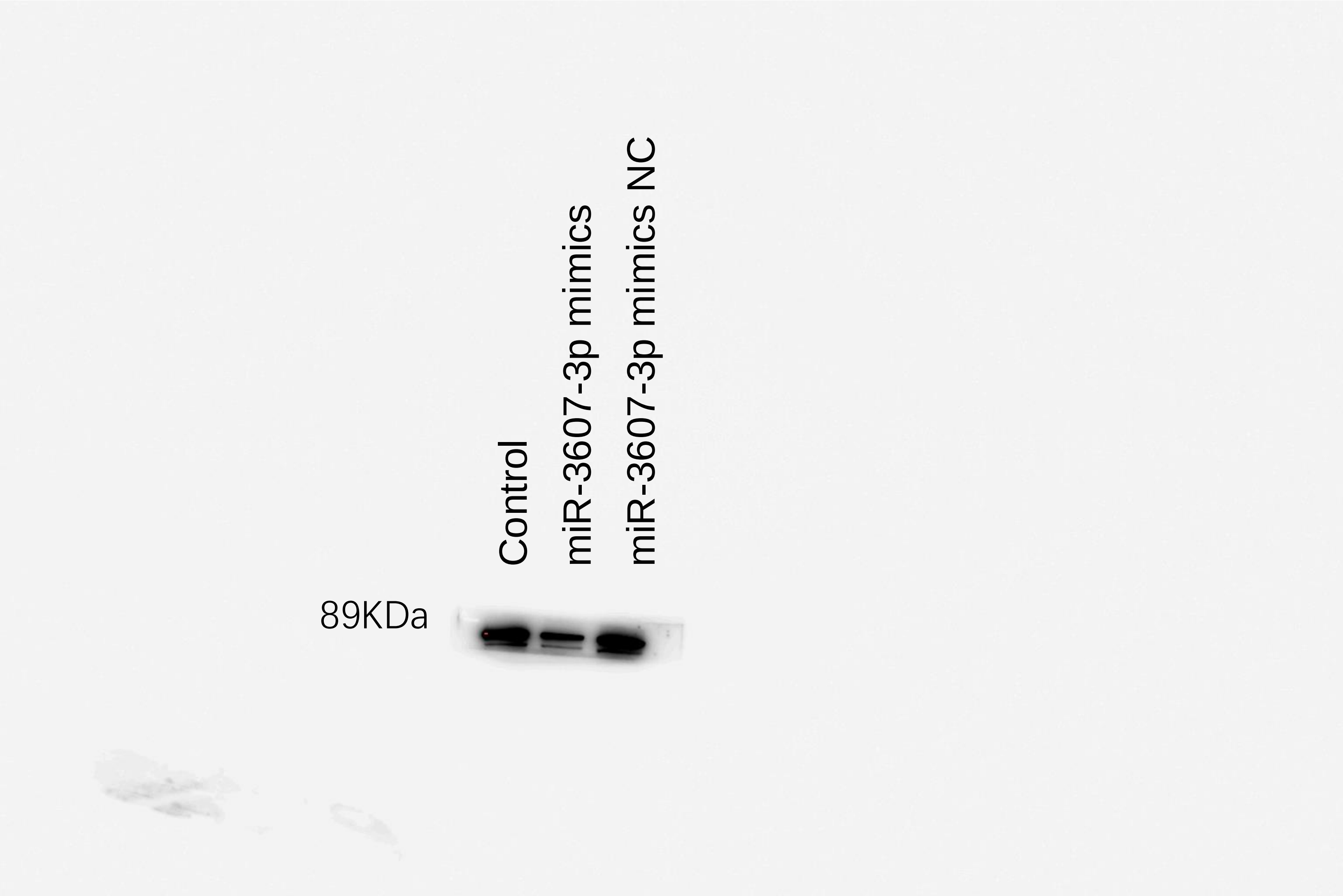

PARP Repeat2

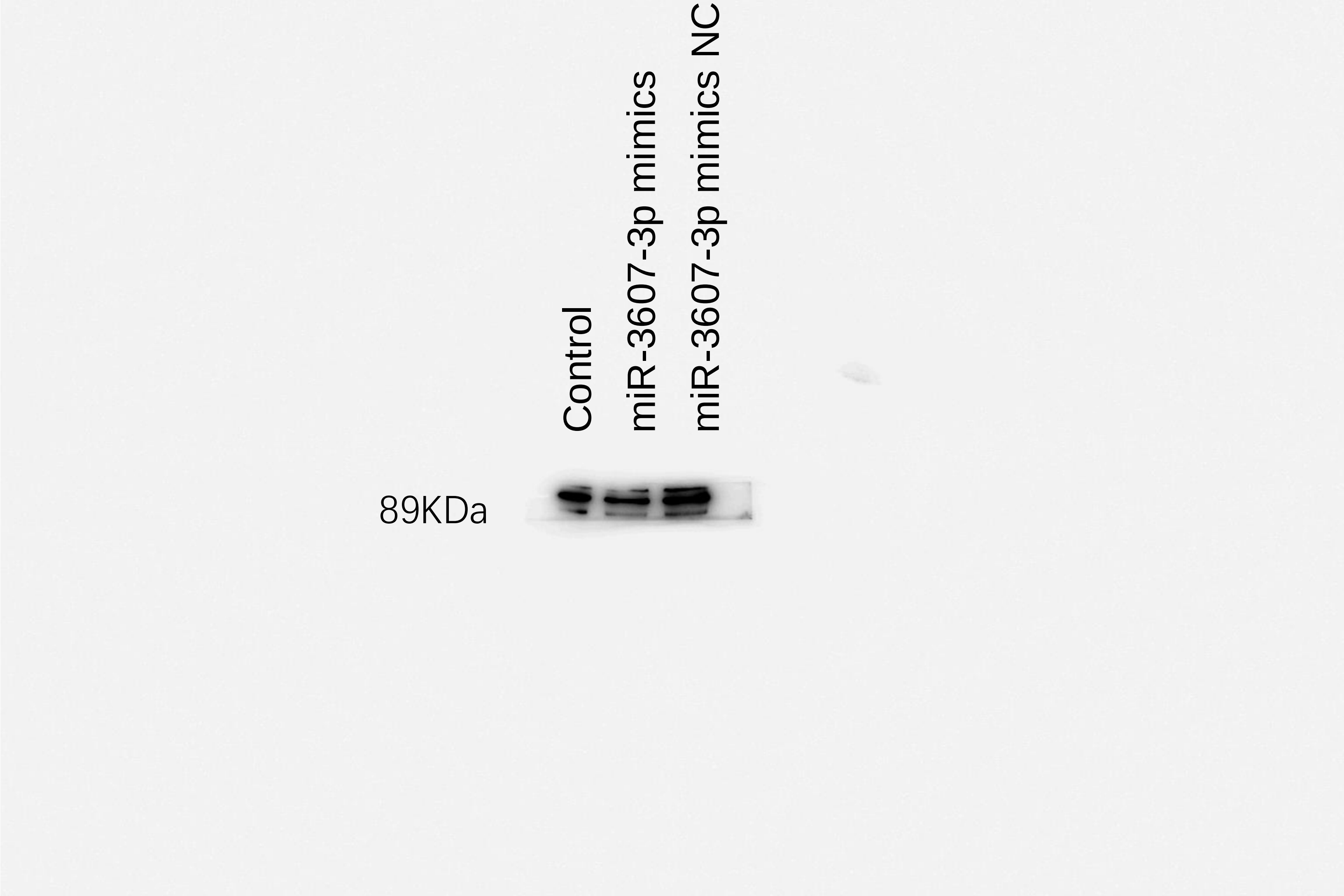

PARP Repeat3

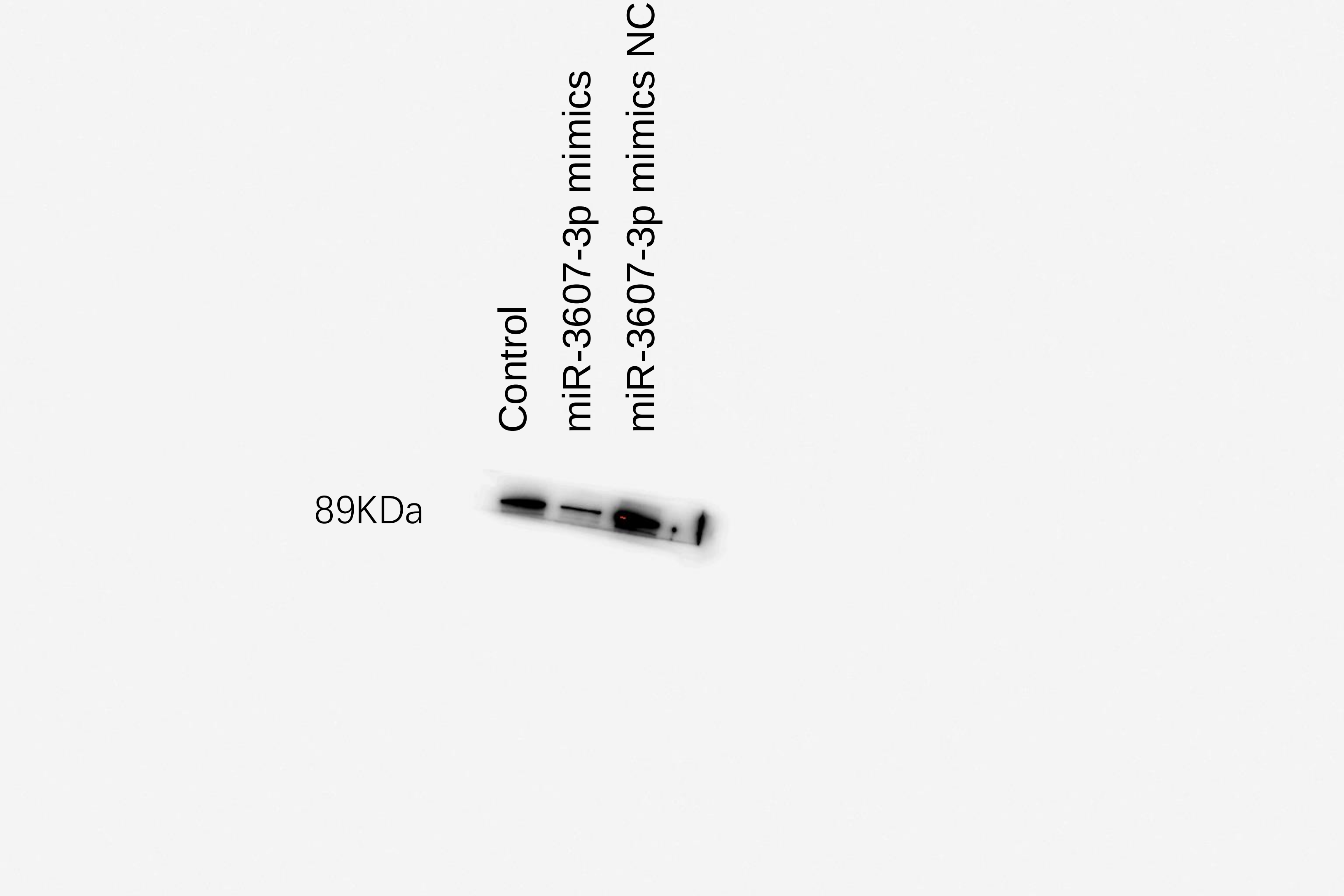

β-actin Repeat1

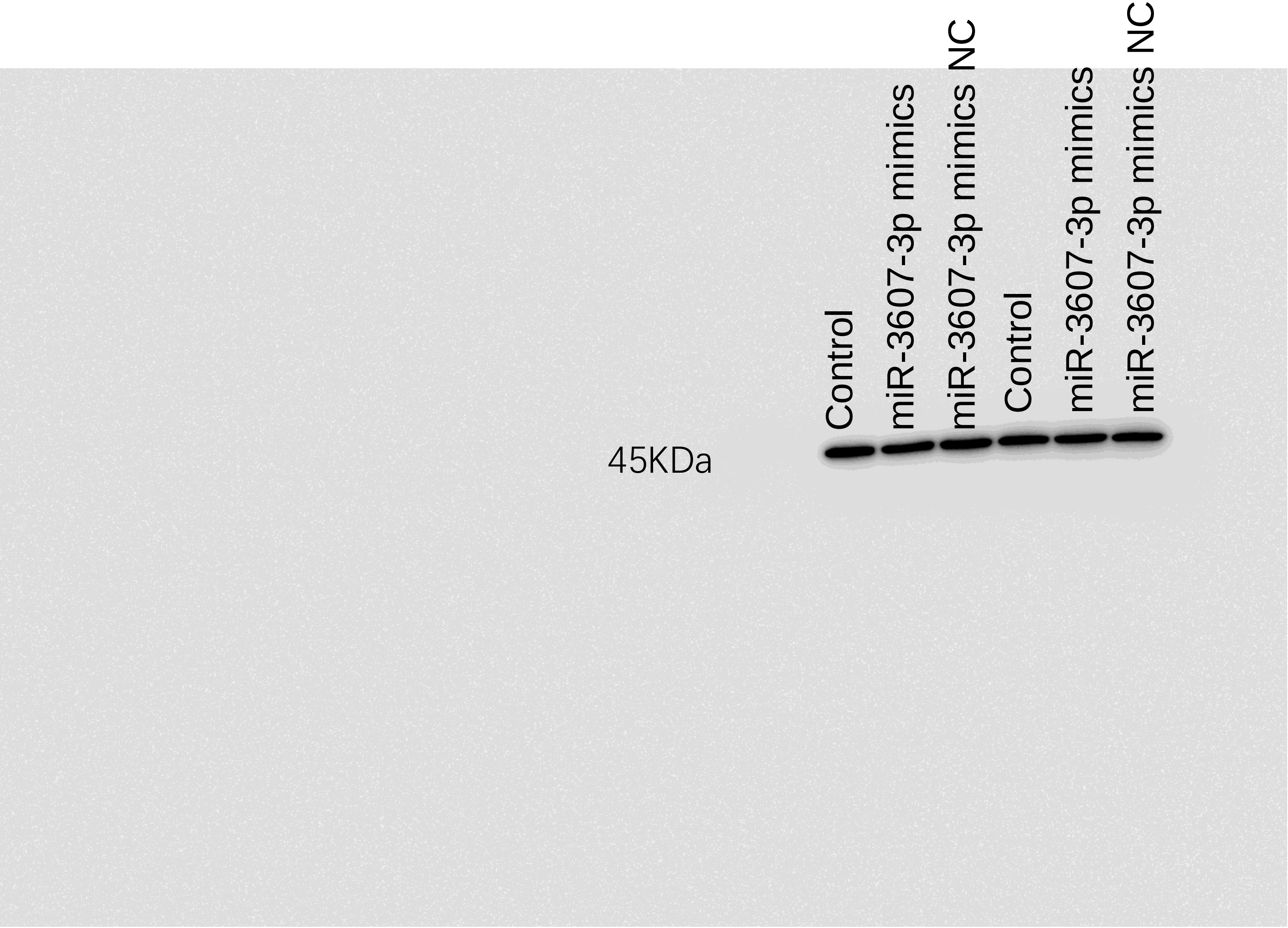

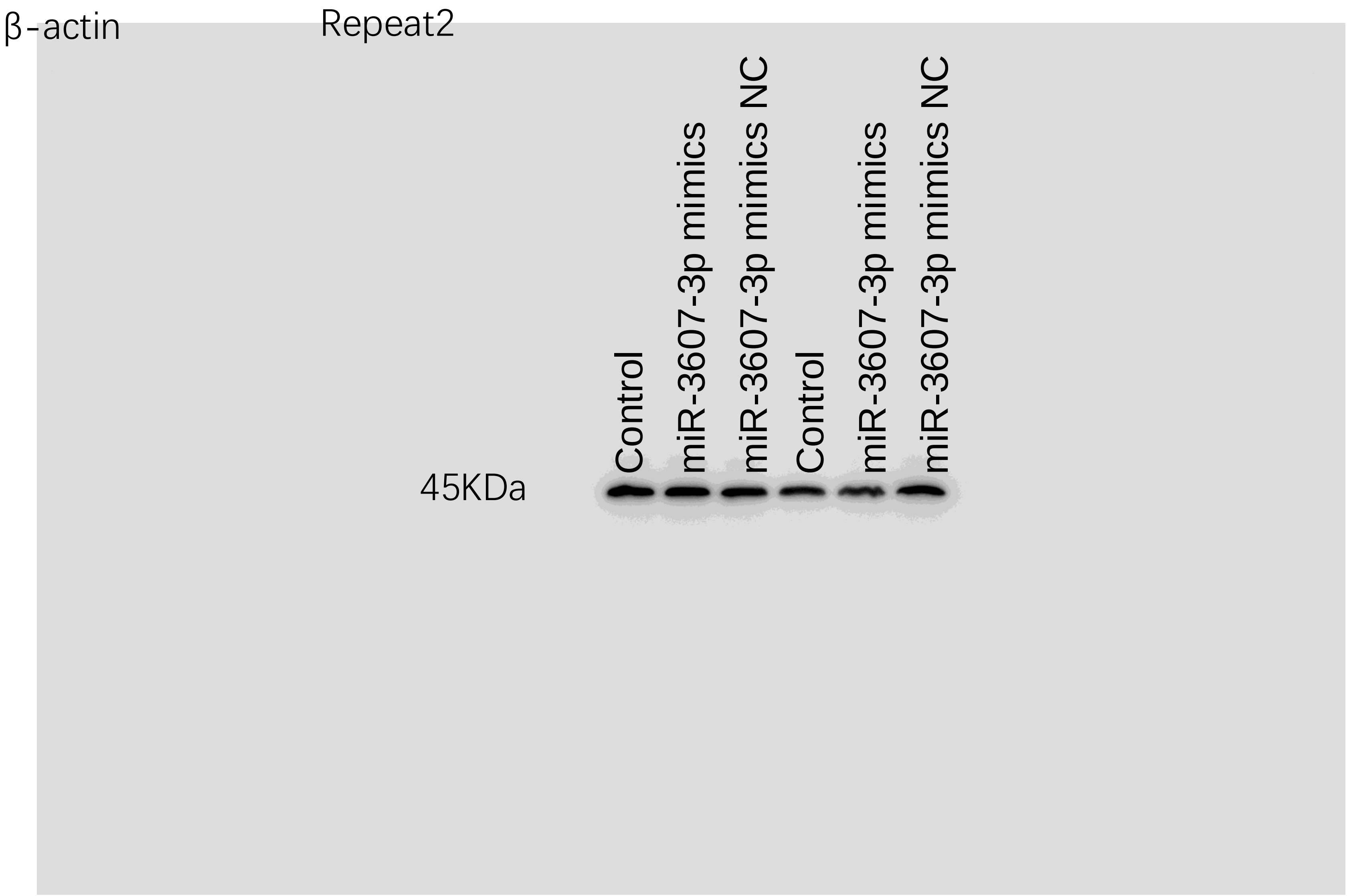
β-actin
Repeat2
Control
miR-3607-3p mimics
miR-3607-3p mimics NC
Control
miR-3607-3p mimics
miR-3607-3p mimics NC
45KDa

β-actin Repeat3

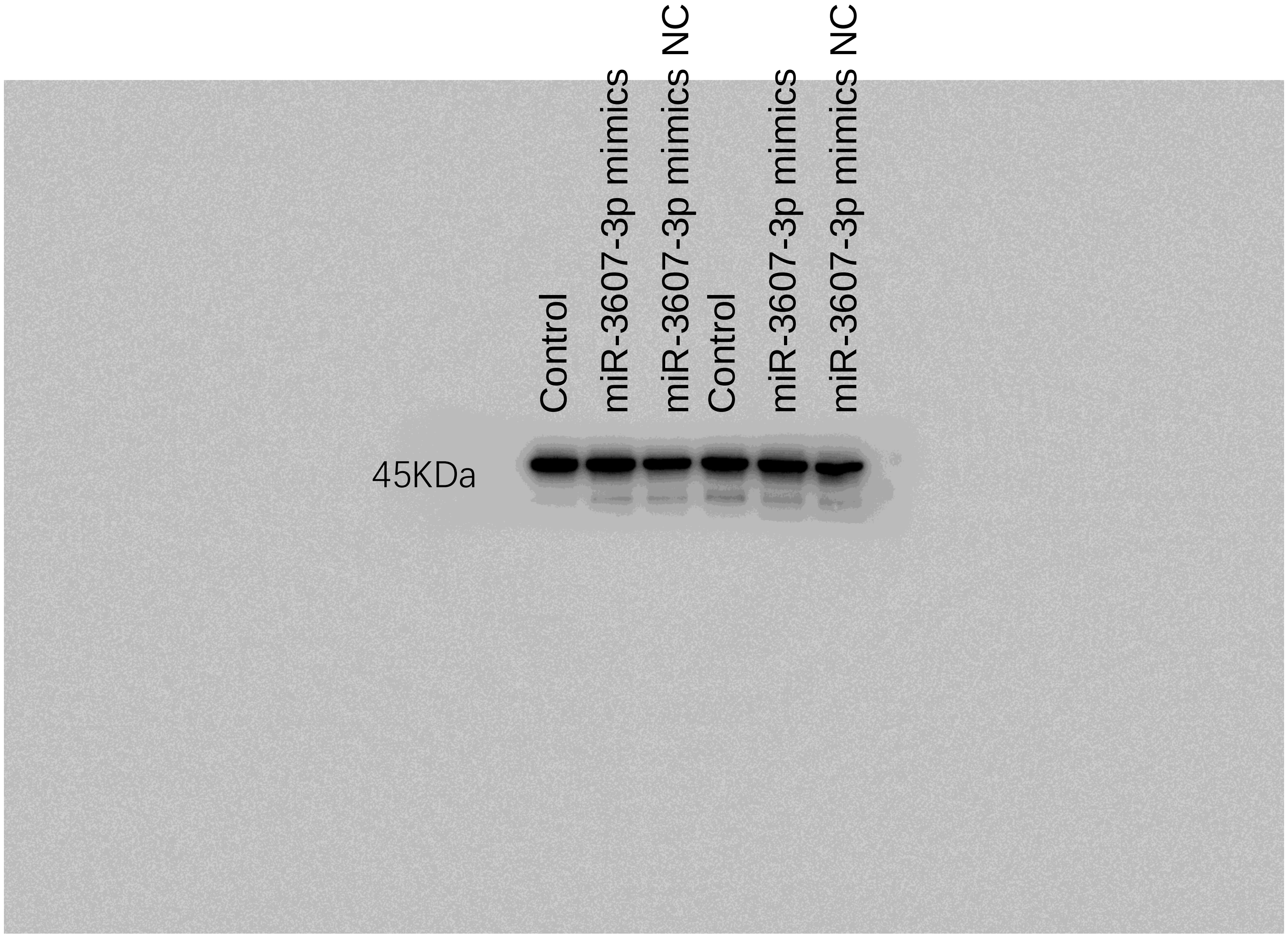

Supplement: Supplementary file 1 — Supplementary Material 1 [file 41598_2025_20112_MOESM1_ESM.pdf]
