# Supplementary figures and images for: Machine learning identifies MiRNA biomarkers and immune mechanisms in active tuberculosis
Source: Sci Rep. 2025 Oct 16;15:36246. doi: 10.1038/s41598-025-20112-8 (PMC12533189; doi:10.1038/s41598-025-20112-8)

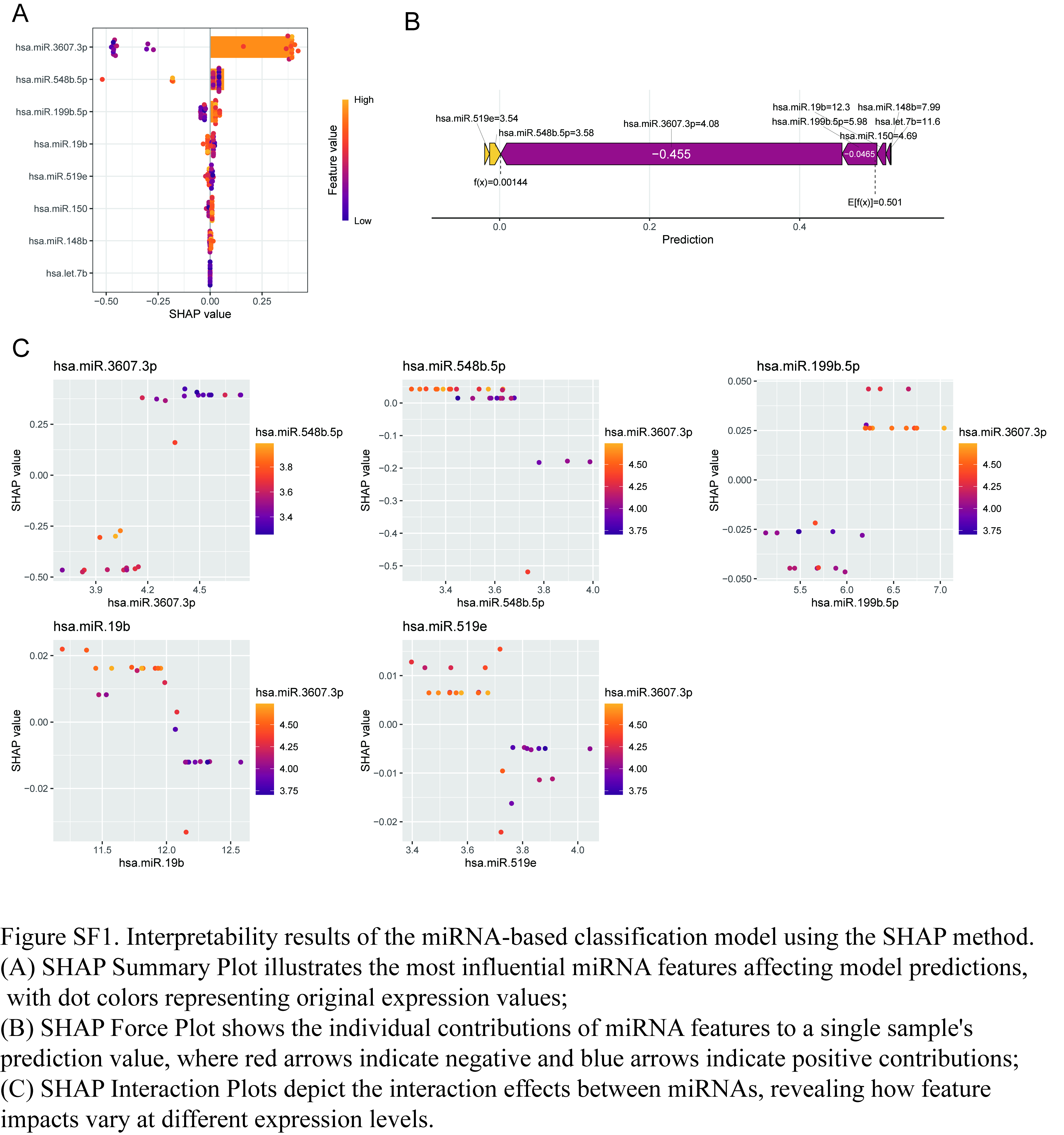

Supplement: Supplementary file 2 — Supplementary Material 2 [file 41598_2025_20112_MOESM2_ESM.tif]

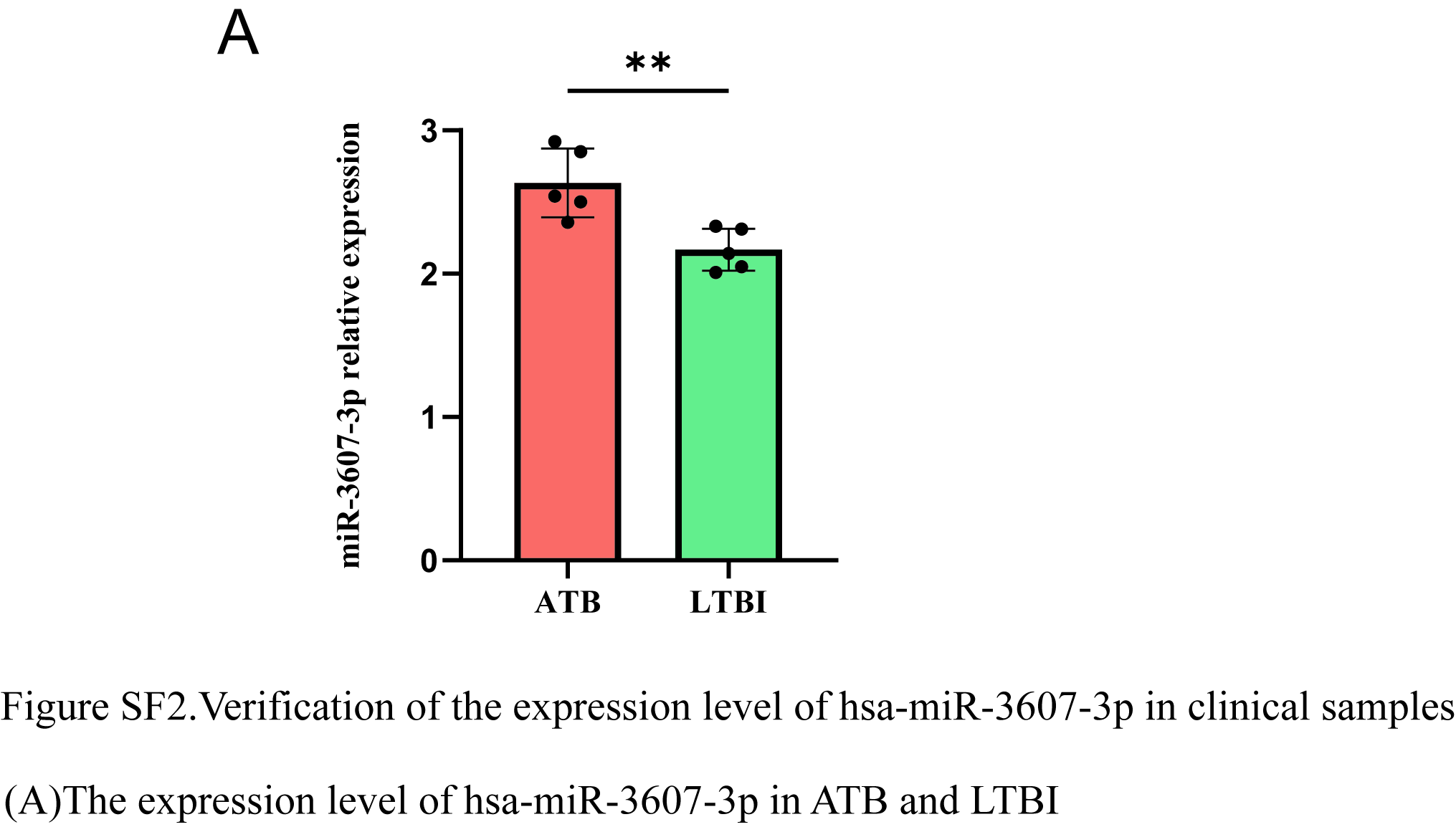

Supplement: Supplementary file 3 — Supplementary Material 3 [file 41598_2025_20112_MOESM3_ESM.tif]
