# Supplementary material for: Machine learning identifies MiRNA biomarkers and immune mechanisms in active tuberculosis
Source: Sci Rep. 2025 Oct 16;15:36246. doi: 10.1038/s41598-025-20112-8 (PMC12533189; doi:10.1038/s41598-025-20112-8)
Supplement: Supplementary file 4 — Supplementary Material 5 [file 41598_2025_20112_MOESM4_ESM.docx]

**Supplementary Table 1. Hyperparameters Used in Each Machine Learning Model**

| Model | Hyperparameters |
| --- | --- |
| XGBoost | learning rate = 0.1, max_depth = 6, n_estimators = 100 |
| Support Vector Machine (SVM) | kernel = RBF, C = 1.0, gamma = 'scale' |
| Random Forest | n_estimators = 100, max_depth = None, max_features = 'sqrt' |
| AdaBoost | n_estimators = 50, learning_rate = 1.0 |
| LogitBoost | n_estimators = 100, learning_rate = 0.1 |
| Partitioning Around Medoid (PAM) | number of clusters = 3, distance = Euclidean |
| Naive Bayes | no tunable hyperparameters |
| Neural Network | hidden_layer_sizes = (100,), activation = 'relu', solver = 'adam', max_iter = 200 |
| Bagged CART | base_estimator = DecisionTreeClassifier(), n_estimators = 100, bootstrap = True |
