# Supplementary material for: Machine learning identifies MiRNA biomarkers and immune mechanisms in active tuberculosis
Source: Sci Rep. 2025 Oct 16;15:36246. doi: 10.1038/s41598-025-20112-8 (PMC12533189; doi:10.1038/s41598-025-20112-8)
Supplement: Supplementary file 5 — Supplementary Material 6 [file 41598_2025_20112_MOESM5_ESM.docx]

**Supplementary Table 2. Model Performance Evaluation Index table**

| Model | Precision | Recall | F1-score |
| --- | --- | --- | --- |
| XGBoost | 1.000 | 0.750 | 0.857 |
| SVM (RBF) | 1.000 | 1.000 | 1.000 |
| Random Forest | 1.000 | 1.000 | 1.000 |
| Bagged CART | 1.000 | 1.000 | 1.000 |
| LogitBoost | 1.000 | 1.000 | 1.000 |
| Partitioning Around Medoid (PAM) | 1.000 | 1.000 | 1.000 |
| Naive Bayes | 1.000 | 1.000 | 1.000 |
| AdaBoost | 1.000 | 1.000 | 1.000 |
| Neural Network | 1.000 | 1.000 | 1.000 |
