# Supplementary material for: Machine learning identifies MiRNA biomarkers and immune mechanisms in active tuberculosis
Source: Sci Rep. 2025 Oct 16;15:36246. doi: 10.1038/s41598-025-20112-8 (PMC12533189; doi:10.1038/s41598-025-20112-8)
Supplement: Supplementary file 6 — Supplementary Material 4 [file 41598_2025_20112_MOESM6_ESM.docx]

| Sample ID | Subject ID | Disease Status | Cell Type | Gender |
| --- | --- | --- | --- | --- |
| GSM1754602 | TB008 | LTBI | granulocytes | female |
| GSM1754603 | TB002 | LTBI | granulocytes | male |
| GSM1754604 | TB001 | LTBI | granulocytes | female |
| GSM1754605 | CTRL008 | TB | monocytes | female |
| GSM1754606 | CTRL003 | TB | monocytes | male |
| GSM1754607 | TB010 | LTBI | monocytes | female |
| GSM1754608 | CTRL005 | TB | granulocytes | male |
| GSM1754609 | CTRL008 | TB | granulocytes | female |
| GSM1754610 | CTRL004 | TB | granulocytes | female |
| GSM1754611 | CTRL002 | TB | granulocytes | female |
| GSM1754612 | CTRL003 | TB | granulocytes | male |
| GSM1754613 | CTRL010 | TB | granulocytes | male |
| GSM1754614 | CTRL004 | TB | monocytes | female |
| GSM1754615 | CTRL009 | TB | granulocytes | female |
| GSM1754616 | CTRL012 | TB | granulocytes | female |
| GSM1754617 | TB011 | LTBI | granulocytes | male |
| GSM1754618 | TB015 | LTBI | granulocytes | male |
| GSM1754619 | TB007 | LTBI | granulocytes | female |
| GSM1754620 | TB005 | LTBI | granulocytes | female |
| GSM1754621 | CTRL005 | TB | monocytes | male |
| GSM1754622 | CTRL002 | TB | monocytes | female |
| GSM1754623 | CTRL010 | TB | monocytes | male |
| GSM1754625 | CTRL009 | TB | monocytes | female |
| GSM1754626 | CTRL012 | TB | monocytes | female |
| GSM1754627 | TB011 | LTBI | monocytes | male |
| GSM1754628 | TB015 | LTBI | monocytes | male |
| GSM1754629 | TB001 | LTBI | monocytes | female |
| GSM1754630 | TB010 | LTBI | granulocytes | female |
| GSM1754631 | TB007 | LTBI | monocytes | female |
| GSM1754632 | TB005 | LTBI | monocytes | female |
| GSM1754633 | TB008 | LTBI | monocytes | female |
| GSM1754635 | TB002 | LTBI | monocytes | male |

**Supplementary Table 3.Sample grouping display**
